# Proposal of a Knowledge Management Model for Complex Systems: Case of the Supervision and Control Subsystem of the Colombian Health System

**DOI:** 10.3390/jmahp12030019

**Published:** 2024-08-21

**Authors:** Fredy G. Rodríguez-Páez, Diego Cabrera-Moya, Jorge Aurelio Herrera-Cuartas

**Affiliations:** 1 Faculty of Economic and Administrative Sciences, Universidad de Bogotá Jorge Tadeo Lozano, Bogotá 110311, Colombia; diegor.cabreram@utadeo.edu.co; 2 Faculty of Natural Sciences and Engineering, Universidad de Bogotá Jorge Tadeo Lozano, Bogotá 110311, Colombia; jorgea.herrerac@utadeo.edu.co

**Keywords:** health policy, health authorities, health sector stewardship and governance, health care coordination and monitoring, knowledge management, complexity, public health systems, sanitary supervision, management audit

## Abstract

Background: Considering regulatory, supervision, and control health policy, an innovative knowledge management model is proposed for the Colombian health system, which is recognized as a complex system. Methods: A model is constructed through a comparative analysis of various theoretical and conceptual frameworks, and an original methodology is proposed based on an analysis of the macroprocesses of the Supervision and Control System (SSC) of the Colombian General Social Security System in Health (SGSSS). After formulating hypotheses and conceptual references, information errors are determined within the different macroprocesses of the SGSSS, including those of governance and the SSC. Results: The risks of generating duplicate, wrong, hidden, or non-existent information arise when the associated regulations need more specificity to be applied in all cases, thus leading to the risk of different interpretations by some actors. In this way, it is possible to hinder the generation of unified information, as there is no clarity as to who is responsible for the generation or creation of certain data. Conclusions: The proposed model is characterized by its flexibility and adaptability, integrating several processes that can be executed simultaneously or cyclically (depending on the system’s needs) and allowing for the generation and feedback of knowledge at different stages, with some processes simultaneously executed to complement each other.

## 1. Introduction

The proper functioning of the SGSSS is required to ensure that the most economically and socially vulnerable people have adequate access to health services [1]. It is essential to have an SSC that guarantees that the interactions among the different actors participating in it contribute to strengthening health equity [2].

Knowledge management has become crucial for organizational success in an increasingly interconnected world reliant on information. This article proposes an innovative knowledge management model (KMM) to enhance collaboration and information exchange among organizations in different sectors, which together form a complex system. Although the model can be applied to various contexts, this study focuses on its implementation in the SSC-SGSSS: an environment that presents unique challenges and opportunities.

The proposed model is based on the following four key components: comprehensive diagnosis, creation of a knowledge culture, capture and organization of knowledge, and external knowledge management. Together, these components provide a comprehensive framework for knowledge management, enabling organizations to identify, capture, organize, and share knowledge effectively within and outside the organization or, in this case, a system as a whole.

Proper knowledge management is fundamental for efficiency and innovation in complex systems. These systems allow for information collection, storage, distribution, and analysis, facilitating decision-making and problem-solving. Proposing models to manage knowledge in complex systems is crucial for improving efficiency, reducing redundancy, and fostering collaboration [3].

However, formal proposals in the related academic literature remain limited. Often, these models need to address the full complexity and dynamics of complex systems, which can result in ineffective knowledge management. Therefore, developing and proposing new models to address these limitations and improve knowledge management in complex systems is essential.

As stated earlier, this article proposes a KMM involving public/official and private organizations to improve collaboration and information exchange. It emphasizes that the SCS-SGSSS behaves as a complex system. By implementing this model, we aim to improve the quality and accessibility of the system’s services.

We hope that this study contributes to the existing literature on knowledge management and provides professionals and academics with a valuable tool to improve collaboration and information exchange in their organizations. Our model has the potential to foster innovation and continuous improvement, which can ultimately lead to better organizational performance.

### 1.1. Theoretical Background

Complex systems are fundamental concepts in various fields, such as social sciences, biomedicine, biology, and computational biology. They are characterized by their intricate structure, non-linear behaviors, and the presence of numerous interconnected components. The study of complex systems has gained significant attention because of their ability to exhibit emergent properties, self-organization, and adaptability [4,5,6,7].

The foundations of complex systems lie in their capacity to model real-world phenomena, such as social interactions, biological networks, and interconnected systems. These systems are often described as complex adaptive systems, emphasizing their ability to adapt and evolve in response to internal and external stimuli [7,8]. Moreover, applying category theory with a “*topos*” perspective has been recognized as a solid basis for modeling complex systems and their evolution with respect to design processes [9].

In the context of networked systems, such as multi-stage interconnection networks, the characteristics of complex systems are evident, leading to discussions on how these systems possess all the attributes of complex systems and should be managed accordingly [10]. Likewise, the philosophical foundations of complex systems have been explored, proposing an alternative ontological basis for the study of incredibly complex systems [11].

The study of complex systems extends to various domains, such as sports injury rehabilitation, urban infrastructure systems, and interdisciplinary research. In sports injury rehabilitation, the characteristics of complex systems have been recognized, emphasizing the need to clarify these characteristics to improve the practical utility of the complex systems approach [8]. Similarly, in analyzing the disaster resilience capacity of urban infrastructure systems, the complex system is defined by many coupled and interconnected components, a complex internal structure, and a global behavior characterized by uncertainty and non-linearity [12]. Furthermore, interdisciplinary research has adopted complex systems as a general approach to solving complex problems in modern science [13].

The theoretical background of complex systems has also influenced leadership theories. Complex leadership theory emphasizes the shift from individual and controlling visions to visions of organizations as complex adaptive systems that enable the continuous creation and capture of knowledge [14]. In addition, systems theory has been proposed as a general approach to understanding the behavior of systems, providing a multi-disciplinary theoretical basis and a discipline-independent framework [15,16].

The theoretical background of complex systems encompasses their interdisciplinary nature, adaptability, and emergent properties. These systems serve as a framework for understanding and modeling a wide range of phenomena, from social interactions to urban infrastructure, and have implications for leadership, research methodologies, and the study of real-world systems [8,9].

#### 1.1.1. Health Systems as Complex Systems

Health systems are complex entities governed by non-linear interaction laws, self-organization, and emergent phenomena [17]. They are characterized by a combination of people, processes, and products and are increasingly dependent on information technology and knowledge. The uncertainties associated with the human-centered aspects of these systems make them particularly complex [18]. Health systems are also influenced by language, structure, logic, and social order, which can lead to unpredictable developmental failure [19].

Furthermore, sustainable outcomes in community health systems can be achieved through complex adaptive system behaviors, and planners and practitioners need to understand the complexity of the context and make reasonable assumptions regarding the impact of interventions [20].

Like any system, health systems require supervision and control schemes to achieve their objectives. A range of studies have explored management control systems in the context of healthcare. Herzlinger et al. found that such systems can lead to motivational changes in staff and improve production characteristics in health centers [21]; Cunningham emphasized the role of management control systems in evaluating performance, planning, and coordinating activities in the British National Health System (NHS) [22]; Saltman highlighted the need for improved management control practices in publicly planned healthcare systems, using the Finnish system as a case study [23]; Kotzian identified the role of agency problems and the potential for external control, such as government intervention, in addressing these issues [24]; and Reginato drew conclusions about the growing need to modernize internal control systems and increase managerial accountability and transparency in spending public money [25]. 

The failure of supervision and control in health systems is a complex issue influenced by cultural, resource, and organizational factors. Clements et al. emphasized the need for a better understanding of human interactions in supervision, particularly in developing countries [26]; Stinson highlighted the lack of human and financial support for supervision, as well as the need for a locally appropriate and sustainable strategy [27]; Bradley underscored the importance of policy-level attention to ensure a systematic and structured supervision process, particularly in the context of mid-level cadres [28]; and Veney et al. provided a broader perspective, suggesting that ministries of health can contribute to the failure of supervision through inadequate funding, centralization, and ineffective use of supervision [29]. These studies collectively underscore the need for a comprehensive approach to address the failures of supervision and control in health systems. Lansky reviewed the failure of contemporary quality oversight organizations to respond to the changing healthcare environment, examined the factors that have limited public accountability for healthcare, and proposed a five-part quality oversight system including the development of quality measures, the promulgation of national standards, validation and accreditation, the use of data for purchasing and provider selection, and the use of data for quality improvement [30].

#### 1.1.2. Knowledge Management Systems (KMSs)

KMSs are designed to support and enhance the organizational processes related to creating, storing, retrieving, transferring, and applying knowledge [31,32]. These systems are crucial in capturing, managing, and disseminating organizational knowledge, thus providing a significant competitive advantage [33]. A KMS enables knowledge retrieval, storage, sharing, and publishing [34]. Implementing a KMS is essential for organizations to utilize knowledge effectively, maximizing knowledge-related effectiveness and creating value [35]. Moreover, a KMS is considered a recent phenomenon in the management circle, reflecting the increasing importance of knowledge in organizational strategies [36].

The benefits of a KMS include facilitating the creation, access, and re-use of knowledge, often using advanced technology [37]. In addition, a KMS can help to develop a Lean Culture within an organization by controlling the “*waste of knowledge*” during project life cycles and utilizing this knowledge in subsequent projects [33]. Furthermore, a KMS can be seen as an activity that involves capturing the best practices and knowledge acquired by individuals and storing them in a computer system for future use [38].

However, implementing a KMS requires much work. Gaps may arise when implementing a KMS, and a holistic framework of the “*Knowledge Management Gap*” has been proposed to demonstrate these management gaps [39]. Moreover, the potential drawbacks of family involvement in strategic management processes may impact the effective management of knowledge resources in family firms [40]. Furthermore, very few methodologies address knowledge management issues, indicating a gap in the methodologies available for best practice in knowledge management [41].

KMSs play a vital role in modern organizations by facilitating the effective management and utilization of knowledge. While they offer numerous benefits, their implementation can be challenging, and comprehensive methodologies are needed to address issues related to knowledge management [3,33,37,42].

#### 1.1.3. Context of Knowledge Management and Its Organizational Contribution

In the rapidly evolving landscape of modern business, knowledge management has become increasingly pivotal. This article delves into the multi-faceted aspects of knowledge management and its profound impact on organizational performance. From the role of knowledge in organizations to the complexity of business systems, a comprehensive diagnosis, creating a knowledge culture, capturing and organizing knowledge, and external knowledge management, each section provides an in-depth exploration of its respective topics. The insights presented herein aim to shed light on the intricate dynamics of knowledge management and its potential to drive organizational success.

Building upon the foundation of knowledge management, this study further explores the intricate mechanisms that underpin the successful implementation of knowledge management strategies. It underscores the importance of a comprehensive diagnosis, which serves as a roadmap for identifying critical areas of knowledge, existing gaps, and potential areas for improvement. The role of organizational culture in fostering a conducive environment for knowledge sharing and continuous learning is also highlighted.

As we delve deeper into knowledge management, we also discuss the significance of capturing and organizing knowledge effectively. Advanced technological and organizational tools are proposed to ensure that knowledge is easily accessible and can be searched efficiently. Finally, this article broadens its scope to include external knowledge management, emphasizing the importance of integrating external sources of relevant information into our processes.

This study explores the multi-faceted nature of knowledge management and its critical role in organizational success. It begins by examining the role of knowledge in organizations, highlighting its value as a strategic asset. It then delves into the complexity of business systems, emphasizing the importance of understanding them for effective knowledge management. The need for a comprehensive diagnosis to identify key knowledge areas, gaps, and potential improvements is also discussed, underscoring the significance of creating a knowledge culture that promotes continuous learning and collaboration. 

Furthermore, the necessity of capturing and organizing knowledge effectively using advanced tools is explored. Finally, the scope of this article is expanded to external knowledge management, stressing the importance of integrating relevant external information into organizational processes. Each of these topics contributes to a comprehensive understanding of knowledge management, providing insights into its potential to drive organizational success.

- The Role of Knowledge in Organizations: Knowledge is an invaluable resource for modern businesses. It represents accumulated experience, processed information, and skills developed over time [43,44]. In a world where technology and information flow rapidly, having efficient knowledge management has become a crucial competitive advantage [45,46].
- The Complexity of Business Systems: Business systems comprise various tangible and intangible interconnected elements. These elements—from technological infrastructure to customer relationships—form a complex network influencing the organization’s overall performance. Knowledge management becomes essential to understanding and optimizing complex systems [47,48].
- Comprehensive Diagnosis: In the management model, it is essential to carry out a comprehensive organizational diagnosis to develop practical knowledge. This in-depth analysis will allow us to identify essential knowledge, existing gaps, and potential areas for improvement. The diagnosis ranges from evaluating organizational culture to mapping information flows and identifying key talents [49,50,51].
- Creating a Knowledge Culture: Organizational culture is fundamental to knowledge management. It is vital to foster a culture that values and promotes continuous learning, collaboration, and idea exchange [36]. This is achieved by implementing training and education programs, creating communities of practice, and recognizing knowledge as a strategic asset [52,53].
- Capture and Organization of Knowledge: Adequate knowledge capture and organization are essential for the proposed model. We propose the use of advanced technological and organizational tools, such as databases, document management systems, wikis, or repositories, to ensure that knowledge is easily accessible and can be searched efficiently, thus collecting, storing, and classifying knowledge in an accessible and structured manner [54,55]. This will facilitate its search and re-use, promoting efficiency and innovation in decision-making [56,57].
- External Knowledge Management: The proposed model focuses on the organization’s internal knowledge, additionally including the management of external knowledge. This involves identifying external sources of relevant information, such as academic research, market reports, and industry trends. Integrating this information into our processes allows organizations to make more informed decisions and stay at the forefront of their sectors [58,59,60].

This study provides a comprehensive overview of knowledge management and its organizational contributions. It aims to serve as a valuable resource for those seeking to understand its complexities and potential to drive organizational success.

### 1.2. Research Question

What are the benefits of having an adequate KMS to optimize the SSC processes of the SGSSS, as an open and complex system that brings together different types of organizations?

## 2. Materials and Methods

We propose to adapt a KMS model whose characteristics, components, and phases obey a series of hypotheses, referents, and stages.

First, based on the scientific literature, hypotheses about the importance of having an adequate KMS were developed. These were complemented with references from Colombia and the proposal of processes and stages, similar to those carried out by consulting companies for their clients.

Second, a survey of the processes of the SSC-SGSSS was carried out through the consensus of experts from the work team who identified, for each macroprocess, its objective, a brief description of the information that enters the process and its usefulness, and a synthesis of the procedural description. The work team advanced the definition of errors and inconveniences generated within the SSC-SGSSS, such as consequence of inconsistencies in information that may give rise to duplicate information; errors associated with erroneous, non-existent information caused by the absence of data that should exist within the process; and hidden information or information that is not available to certain actors to whom it should be available. Subsequently, an in-depth analysis of the problem was carried out, in terms of the waste or unnecessary consumption of resources and time caused by the shortcomings described above, which was analytically proposed by the work team to finally present a small synthesis of the ideal situation that would be achieved if these errors in knowledge management could be resolved.

Third, if these errors in knowledge management could be solved, a small synthesis of the ideal situation could be achieved by resolving these errors in knowledge management, which is the basis for the proposed model, its components, and the phases of its implementation.

In order to facilitate the reading of this article, a list of abbreviations was prepared. The abbreviations are organized in alphabetical order, with their meaning first in English and, after the “/” sign, the official name (or the usual term used in Colombia) in Spanish is provided. In the case of public organizations or legal concepts included only in diagrams or in Supplementary Materials a URL is also included (see Table 1).

## 3. Results

The first subsection presents the hypotheses and the proposed methodology with its references and stages prior to the development of the model; the second subsection provides the results of the analysis of the macroprocesses; and the third subsection introduces the proposed model, including its components and implementation phases.

### 3.1. Hypotheses, References, and Stages

The hypotheses and the proposed methodology with its references and stages prior to the development of the model are presented below.

#### 3.1.1. Hypotheses

A practical KMS is crucial for optimizing processes in a complex system that brings together different organizations [42,61,62,63,64,65]. It allows organizations and complex systems to capture, store, organize, and disseminate knowledge, leading to improved performance, innovation, and competitive advantage [66,67].

One of the main benefits of a KMS is its ability to facilitate knowledge sharing and collaboration among the organizations within a complex system [66,68,69]. By providing a centralized platform for storing and accessing knowledge, organizations can easily share best practices, lessons learned, and expertise, which improves decision-making and problem-solving [63,64,70]. Additionally, a KMS helps organizations capture and retain tacit knowledge, which is often difficult to transfer and can be lost when employees leave the organization [42].

Furthermore, a KMS enables organizations to optimize processes by providing access to relevant and up-to-date information [61,62,71]. This allows organizations to make informed decisions, avoid duplication of efforts, and reduce errors [68,69]. By leveraging the knowledge and expertise available within the system, organizations can streamline their operations, improve efficiency, and achieve cost savings [71,72].

A KMS is a complex system that brings together different organizations, promoting innovation and driving continuous improvement [61,65,73]. By capturing and organizing knowledge from various sources, organizations can identify new opportunities, generate novel ideas, and foster a culture of innovation [62,74,75]. Moreover, a KMS enables organizations to learn from past experiences and leverage existing knowledge to develop new products, services, and processes [61,65,74,76].

KMSs are essential for optimizing processes in a complex system that brings together different organizations. They facilitate knowledge sharing, provide access to relevant information, promote innovation, and drive continuous improvement. Leveraging the power of knowledge, organizations can enhance their performance, gain competitive advantage, and achieve sustainable growth [42,76,77].

#### 3.1.2. References for the Proposed Model

The innovative and comparative character of the model requested in the Consultancy—which gave rise to the proposal whose results are reported in this article—allowed the development of a unique proposal that, although starting from the basic structure of knowledge management models reported in the literature by different international consulting firms and academics, comprises components and flows within a flexible process that allows the particularities of a complex system, such as the SSC-SGSSS in which the proposal is developed, to be addressed.

The theoretical foundation built at the beginning of the writing this study highlights that a holistic and innovative knowledge management model must simultaneously integrate different processes. This means that, although the proposal contains sequential phases for its implementation, once the model defined for the methodology is implemented, these are executed simultaneously or cyclically, depending on the system’s needs to achieve the objectives defined in each stage. The comparative character defined in the objective also allows for the analysis of related sources in the section associated with the theoretical and conceptual frameworks presented at the beginning of this document, resulting in a comparative proposal by the work team of this Consultancy, which takes the best practices from each of the analyzed sources to define a novel methodology. 

As part of this study, and to complement the analyzed references stated in previous paragraphs, the document defined by the Knowledge Management Directorate of the Administrative Department of Public Function of Colombia in 2020 was also analyzed, titled “Guide for the Implementation of knowledge management and innovation within the framework of the integrated planning and management model (MIPG)” [78], attesting to the fact that some of the entities that interact in the SGSSS are of an official and public nature. 

The particularities highlighted above endow the proposal with the characteristics defined in the objective. This is achieved not only in response to the simultaneous process of both original design and adaptation by the team but also by taking into account national and international references that enable the comparative character of this methodology. Thus, the method simultaneously takes advantage of different experiences from external references and the experience of the work team to consolidate this approach.

#### 3.1.3. Stages of the Knowledge Management Model

The process of analyzing proposals available in the business world related to consultancy studies associated with KMSs allowed for the synthesis of the processes and stages that have been advanced to design models that are adjusted to the needs of clients. It is essential to note that, in this study, the sequence presented below was considered to elaborate the defined innovative proposal.

- Preparation Stage: The first stage of the process involves obtaining the directors’ support and commitment, identifying the problem, and developing an appropriate knowledge management strategy for the organization.
- Knowledge Capture and Collection Stage: The second stage of the process involves identifying and collecting the explicit or tacit knowledge that the organization requires using different methods and tools.
- Distribution and Application of Knowledge Stage: The third stage involves disseminating, transferring, and effectively and efficiently using knowledge in the organization through mechanisms and technologies that facilitate access to and delivery of knowledge.
- Re-use or Recycling of Knowledge Stage: The fourth stage focuses on leveraging and applying existing knowledge effectively and efficiently in the organization by identifying, adapting, and re-using previously acquired knowledge to address new challenges or situations.
- Permanence and Use of Knowledge Stage: The fifth and final stage of the process focuses on ensuring that the captured and generated knowledge is used effectively and sustainably in the organization through maintenance, updating, promotion, monitoring, and continuous improvement in knowledge activities.

#### 3.1.4. Context of the Health System in Colombia

The SSC for the provision of individual care services caused by general illness or maternity is organized by the SGSSS [79,80,81,82] based on the regulated market, with the participation of both public and private organizations, the latter constituting the majority.

All residents must join an Entidad Promotora de Salud (EPS) [83], which guarantees access and payment for all care required in an Institución Prestadora de Salud (IPS) [84] network. Families with the ability to pay (basically workers and pensioners) periodically contribute a percentage of income to the Régimen Contributivo (RC) [85], while poor families are subsidized by the State in the Régimen Subsidiado (RS) [86]. With permanent mobility between RC and RS, families can also periodically change EPS, according to their needs [87].

When using the services, users must pay a co-payment or moderation fee, the value of which is determined in accordance with their ability to pay [88]. The contributions and subsidies are received by Administradora de los Recursos del Sistema General de Seguridad Social en Salud (ADRES) [89], which transfers a Unidad de Pago por Capitación (UPC) [90] to the EPS to cover the value of the care contemplated in the Plan de Beneficios en Salud (PBS) [91]. Additionally, ADRES transfers a value of Presupuestos Máximos en Salud (PMS) [92] to the EPS in order to cover care financed within the PBS. The Ministerio de Salud y Protección Social (MSPS) [93] periodically defines the PBS, the UPC value, and the PMS. Additionally, the Cuenta de Alto Costo (CAC) [94] is an organization that proposes adjustments in the transfer of resources among EPSs because of the concentration of certain health risks.

The EPS and IPS must comply with financial, technical, and administrative requirements to be able to operate, including requirements established by the MSPS and monitored by the Superintendencia Nacional de Salud (SNS) [95] and other public organizations such as Unidad de Gestión Pensional y Parafiscales (UGPP) [96]; other superintendencies such as Superintendencia de Industria y Comercio (Supercomercio) [97], Superintendencia de Sociedades (Supersociedades) [98], and Superintendencia Financiera de Colombia (Superfinanciera) [99]; and other national entities such as the Contraloría General de la República (CGR) [100] and the Procuraduría General de la Nación (PGN) [101]. Furthermore, compliance monitoring is conducted with the guarantee of the Derecho Fundamental a la Salud (DFS) such as the Defensoría del Pueblo (DFP) [102], the judicial system [103], and the Corte Constitucional (CC) [104].

The health technologies that are marketed in Colombia are evaluated for their benefit and economic and budgetary impacts by the Instituto de Evaluación Tecnológica en Salud (IETS) [105] and authorized by Instituto Nacional de Vigilancia de Medicamentos y Alimentos (INVIMA) [106]. On the other hand, the health situation is monitored by the Instituto Nacional de Salud (INS) [107] and its Observatorio Nacional de Salud (ONS) [108]. Users are organized into user or patient associations [109] in order to manage improvements in quality of care or DFS compliance. 

Services resulting from traffic accidents are covered by the Seguro Obligatorio de Accidentes de Tránsito (SOAT) [110] under the responsibility of each motor vehicle owner. Public health services in Plan de Intervenciones Colectivas (PIC) [111] are financed under the responsibility of each municipality or district, and care resulting from emergencies and disasters is covered by the Sistema Nacional de Gestión del Riesgo de Desastres (SNGRD) [112]. There are populations that are served by excepted regimes, such as the military and police forces, teachers in the public system, and workers of the Colombian oil company 

In Colombia—a country of 52 million inhabitants with a life expectancy at birth for 2023 of 77.23 years [113]—public spending on health represented 6.53% of its GDP and 19.47% of total public spending by 2020, while out-of-pocket spending in health accounted for 13.59% of total health spending [114]. It is classified as upper-middle-income and had a health expenditure of 9.02% for the year 2021 [115].

Compared with other upper-middle-income Latin American and Caribbean countries, this current expenditure as a percentage of GDP is surpassed by Cuba (13.78%), Brazil (9.89%), El Salvador (9.71%), Argentina (9.70%), and Chile (9.34%). On the contrary, it surpasses other countries like Ecuador (8.28%), Paraguay (8.03%), Costa Rica (7.56%), and Mexico (6.07%). Likewise, it is low compared with OECD countries (13.35%), but higher than the group of upper-middle-income countries (5.82%) [116]. Comparatively, for the period 2010–2017, per capita spending on health in Colombia, as a percentage of GDP (PPP USD 2017), was 1114 while, for Latin America and the Caribbean, on average, this figure was 1025, and the OECD average was 3994 [117].

The increase in health spending is associated with a longer life expectancy and aging of the population, an increase in chronic non-communicable diseases, technological advances in health, higher expectations of the population, and unhealthy lifestyle habits that increase the demand and use of services [117,118,119,120,121].

According to the WHO, achieving universal health and guaranteeing the right to health depend on the accessibility, availability, acceptability, and quality of health personnel. Furthermore, the functioning and resilience of health systems depend on the availability of such health personnel, as health workers play an essential role in ensuring access and improving the health of the population [122].

Following the development of medical technologies is crucial for effective healthcare management and improved patient outcomes. It enables preparedness and planning for the updating of healthcare systems, helping decision-makers to identify promising technologies early [123]. Medical devices are indispensable for performing medical services, and their importance has become a priority at institutional and national levels [124]. Emerging technologies such as wearable devices, artificial intelligence, and telemedicine have shown promise in revolutionizing healthcare and addressing challenges such as emerging diseases and staff shortages [125]. Medical technology has a significant impact on prevention, diagnostics, therapy, and rehabilitation, with fields like imaging technology and minimally invasive surgery dominating future developments. Moreover, medical technology enhances the cost-effectiveness of healthcare and is an economic factor with fast innovation cycles and high growth potential [126].

### 3.2. Results of the Analysis of the Macroprocesses

A summary of the main diagnosis for each macroprocess prepared by the project team is provided below. Emphasis is placed on macroprocess diagrams and findings on errors in the information that cause the waste or unnecessary consumption of resources. Further details can be found in Supplementary Materials, S1 to S5.

#### 3.2.1. Macroprocess Diagrams

**Figure 1:**
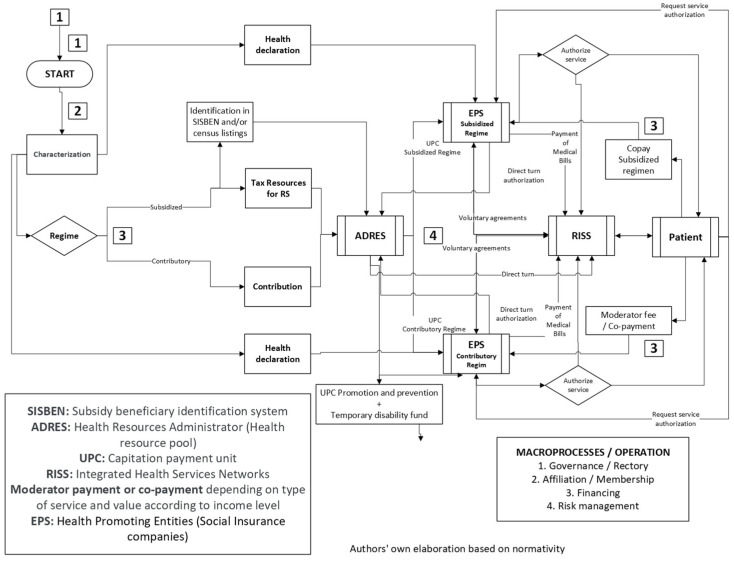
Operation macroprocesses of the Colombian SGSSS.

The project team identified the following five macroprocesses associated with the SSC-SGSSS: governance, affiliation, financing, risk management, and surveillance. The macroprocesses for the operation of the SGSSS are shown in Figure 1.

**Figure 2:**
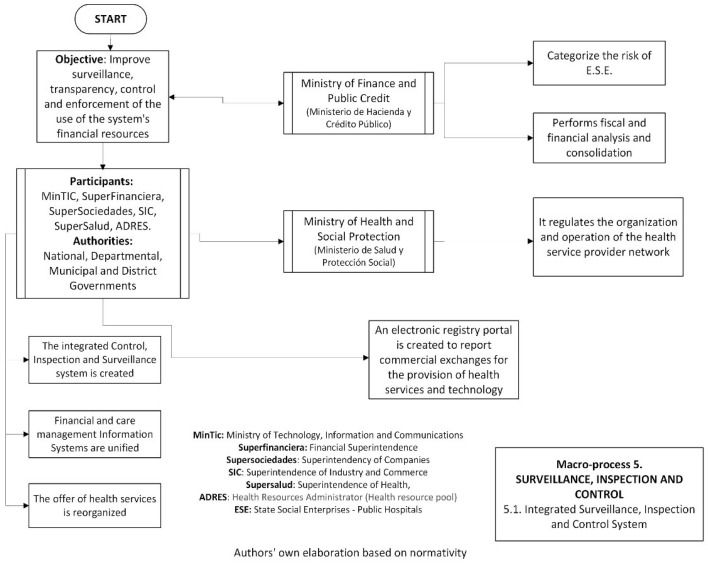
Integrated macroprocess components of surveillance, inspection, and control.

The integrated macroprocess components, objectives, and functions of the main entities of surveillance, inspection, and control are shown in Figure 2. 

**Figure 3:**
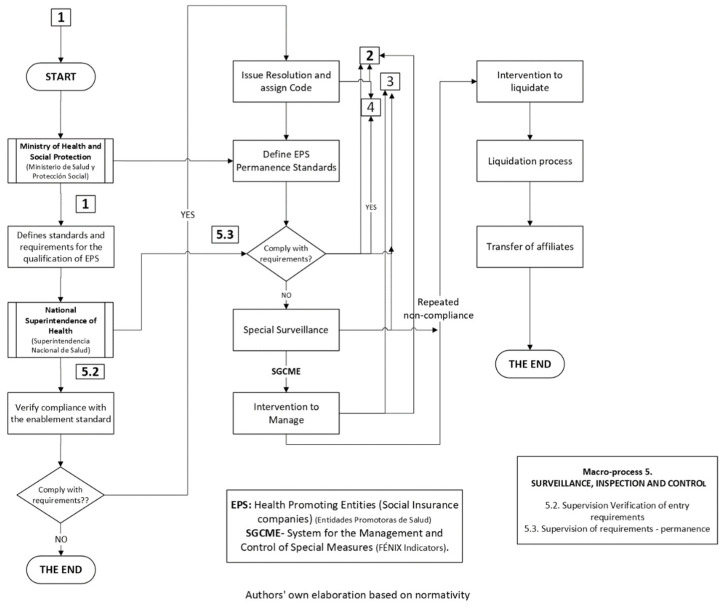
Supervision of entry, permanence, and non-compliance requirements of the EPS in the SGSSS of Colombia.

Figure 3 shows the verification of the entry and permanence requirements of the EPS, as well as their intervention and liquidation of operations in the event of non-compliance.

**Figure 4:**
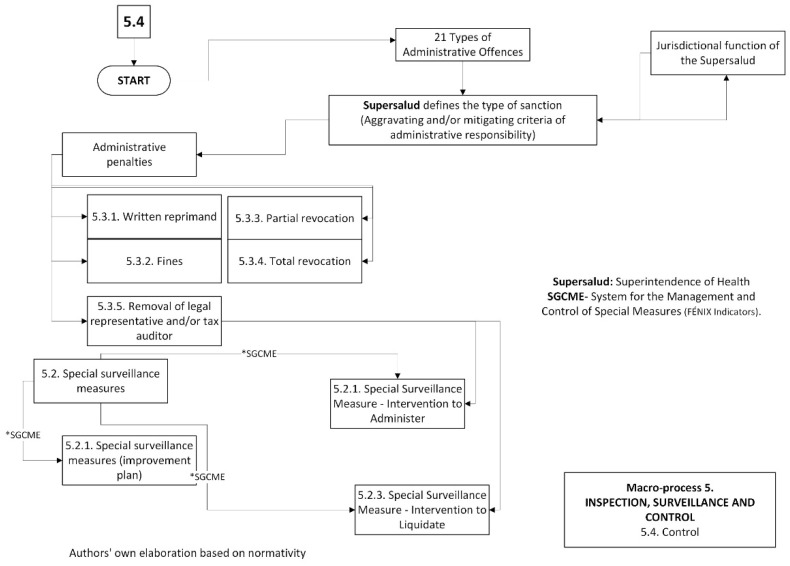
Control actions against the EPS in the SGSSS of Colombia.

Finally, Figure 4 details the control actions taken against the EPS in the event of non-compliance with the requirements or administrative infractions.

#### 3.2.2. Synthesis of Errors in Information within Each SSC-SGSSS Macroprocess

The main findings for macroprocesses 1 of governance/stewardship/regulation and 5 of surveillance, inspection, and control in terms of duplicate, hidden, erroneous, or non-existent information and the impact on the greater resource consumption for the SSC-SGSSS are presented below. The details and findings for the other analyzed macroprocesses are provided in Supplementry Materials, S1 to S5.

Errors identified related to hidden, erroneous, or non-existent duplication of information, as well as the existence of unnecessary or excessive consumption of time or other resources caused by such errors in the macroprocesses of governance, stewardship, and regulation of the system, are detailed in Table 2.

Errors due to duplicate information in the SSC-SGSSS macroprocess of Colombia are presented in Table 3, which are classified into two categories as follows: the first by duplicity in the same source and the second by duplicity in sources. In the first case, the system is fed information that may have been duplicated from the source belonging to other macroprocesses of the SGSSS. A second category involves the duplication of information originating in this same macroprocess, which arises when different sources of information that have yet to be refined or unified and are part of different stages are used. Associated with the portfolio, a need for more agreement was also evident in the periods to which the publication of this information corresponds and/or the periodicity with which the entities issue it. For example, some reports are issued quarterly, such as the one corresponding to the Ministry of Health, while the equivalent by the Superintendence of Health is issued monthly. This leads to a risk of duplicate or erroneous information, which is difficult to detect because of a difference in categories and, possibly, in the format and structure of the consolidated reports.

In the IVC macroprocess, there is a risk of wrong, hidden, or non-existent information that can be generated via the same two situations explained above. These causes stated in the previous section involve, among others, the use of different sources of origin access at different times. This makes it possible for transformation or reporting of the same information to have been carried out by different entities, originating in different databases that are different from each other but which should be exactly the same. Furthermore, changes in the information or category of users that are not purified generates additional records, instead of resulting in unification, and the existence of different control entities that require the same information from different sources that have not been filtered, leading to financial and/or accounting information that presents errors, unreconciled differences, and differences in the frequency with which the information is consolidated in the sources, thus making it incomparable. In addition to the above, other situations could be evidenced as causing these errors, such as those presented in Table 4.

These risks include generating wrong, hidden, or non-existent information when the associated regulations lack sufficient specificity to be applied in all cases and, therefore, allow different interpretations by some of the actors. This possibility also prevents the generation of unified information given that, in some cases, there is also no clarity regarding the individual actor responsible for its generation or creation.

The unnecessary or excessive time and/or resource consumption caused by the identified errors implies the significant consumption of resources and time to correct the errors mentioned above. In most cases, this additional and unnecessary consumption is associated with aspects such as the time necessary to purify the databases and correct erroneous results, as well as the use of additional human resources associated with clarification and specification of the information.

In cases where different actors need to correct this information, there are no unified processes to achieve this; therefore, error correction can be addressed simultaneously or asynchronously from different instances to achieve the same result. Even so, it does not ensure an adequate and definitive correction as there are no unified procedures, nor are there alerts that identify the information that has already been corrected or still needs to be corrected, which can generate re-processes that do not ensure this formalization.

These situations make the procedures associated with the SSC-SGSSS difficult because of the existence of duplicate, erroneous, and untimely information that, when added to the weaknesses of the supervision process, generates decision-making that is late and/or based on unreliable facts and data.

### 3.3. Proposed Model for the Management of Knowledge to the Supervision and Control Subsystem of the Colombian General Social Security System in Health (SSC-SGSSS)

The model proposed in this article integrates several processes that can be executed simultaneously or cyclically, depending on the system’s needs. This allows for the generation and feedback of knowledge at different stages, while some stages can be executed simultaneously to complement each other. 

For this proposal, a comparative analysis of various sources—including theoretical and conceptual frameworks—was carried out, and a proprietary methodology was developed based on the best practices identified. As part of this analysis, a document from the Knowledge Management Directorate of the Administrative Department of Public Function of Colombia, titled “Guide for the implementation of knowledge management and innovation within the framework of the integrated planning and management model (MIPG)” [78], was also examined. 

The proposal stands out for its original design and ability to adapt to the system’s needs. It also considers national and international references and takes advantage of external references and the experiences of the work team. This study presents an innovative and comparative approach to knowledge management through the use of a flexible model that can adapt to complex systems.

#### 3.3.1. Components of the Proposed Model

The nine different components of the proposed macroprocesses are knowledge management strategy, organizational culture, knowledge management subprocesses, information technology, human resources, technology and transactional databases, measurement and evaluation, and organizational learning, which are further detailed in Table 5.

#### 3.3.2. Phases for the Implementation and Execution of the Model

**Figure 5:**
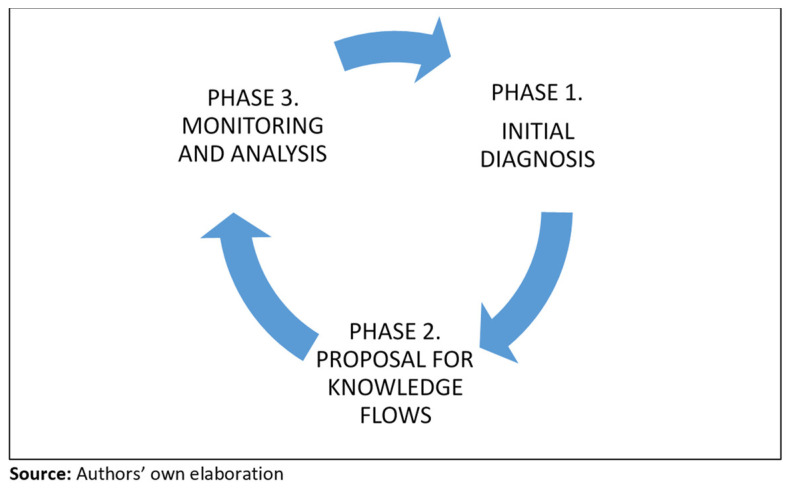
Cycle for the implementation and execution of the KMS model.

This section proposes three phases that must be executed sequentially during the initial implementation process. However, once the management system is in operation, these should be executed simultaneously and cyclically for optimal maintenance (Figure 5).

In order to understand the correspondence of this proposed sequence of phases, in comparison with the five stages belonging to the traditional models mentioned above, we define the relationship between the proposed phases and the objective of each one with the traditional stages in Table 6 in order to facilitate future understanding of the possible interactions with models of other systems that have already been analyzed from this point of view.

#### 3.3.3. Benefits and Limitations of the Proposed Knowledge Management Model

- Benefits.

The proposed Knowledge Management Model (KMM) presents several significant benefits aimed at optimizing the Supervision and Control Subsystem (SCS) of the Colombian General Social Security System in Health (SGSSS). First and foremost, the model fosters improved collaboration and information sharing among various stakeholders, including public and private entities. By creating a centralized platform for knowledge capture and dissemination, the model enhances decision-making processes and reduces the occurrence of information silos, which often lead to inefficiencies and errors [3].

Moreover, the KMM is designed to be both flexible and adaptive, allowing for simultaneous or cyclic execution of processes as needed by the system. This adaptability ensures that the model can respond to the dynamic nature of the healthcare environment, providing continuous feedback and fostering a culture of ongoing improvement and innovation [127]. Integrating best practices from both national and international references, the model stands to significantly improve the quality and accessibility of healthcare services, ultimately leading to better health outcomes for the population [128]. 

Additionally, the model’s emphasis on a comprehensive diagnostic approach ensures that critical areas of knowledge are identified and addressed, thereby closing existing gaps and optimizing resource utilization. This thorough diagnostic capability not only streamlines operations but also enhances the system’s ability to adapt to new challenges and opportunities [129].

- Limitations.

Despite its robust framework, the proposed KMM is not without limitations. One primary concern is the potential complexity involved in its initial implementation. The requirement for substantial initial investment in terms of time, training, and resources could pose a barrier for some organizations, particularly those with limited budgets or expertise in knowledge management systems [130]. 

Furthermore, the effectiveness of the model is heavily dependent on the active participation and commitment of all stakeholders. Resistance to change and varying levels of engagement among different entities could impede the model’s full potential. Ensuring consistent and widespread adoption across the entire SGSSS requires ongoing effort and strong leadership [131].

Another limitation is the potential risk of data overload. With the centralized collection and management of vast amounts of information, there is a possibility of encountering challenges related to data quality, storage, and retrieval. Effective data governance mechanisms must be put in place to mitigate these risks and ensure the reliability and usability of the captured information [127].

The current limitations related to data integrity highlight the necessity and potential impact of the proposed knowledge management model. By systematically addressing these issues, the model is expected to pave the way for more accurate and reliable reporting, ultimately leading to improved decision-making and healthcare outcomes in Colombia [129].

#### 3.3.4. Evaluation of the Model’s Feasibility

- Implementation Complexity.

The assessment of the feasibility of the proposed knowledge management model (KMM) within the context of the Supervision and Control Subsystem (SCS) of the Colombian General Social Security System in Health (SGSSS) involves several critical factors. One of the primary considerations is its complexity of implementation. Given the comprehensive nature of the model, significant initial investments into training, development programs, and IT infrastructure will be required. A phased implementation approach can mitigate the associated complexity, starting with pilot projects in select regions, which will allow for refinement of the model before its broader deployment [127].

- Resource Availability.

Resource availability—both financial and technical—is another critical factor. Establishing a centralized data repository and advanced analytical tools necessitates substantial financial investments. However, experiences from similar implementations in countries with advanced healthcare systems (e.g., the NHS in the U.K. and Scandinavian health systems) suggest that while the initial costs can be substantial, the long-term benefits in terms of operational efficiency and improved health outcomes often justify these investments [3].

- Stakeholder Engagement.

The success of the KMM heavily relies on the active participation and commitment of all stakeholders, including public and private entities, healthcare providers, and regulatory bodies. Regular workshops, feedback sessions, and training programs are essential to foster a culture of knowledge sharing and continuous improvement. Strong leadership is crucial to drive the adoption of the model, as evidenced by the successful implementation of electronic health record systems in Denmark, in which clear communication and leadership were reported to be key factors [130].

- Comparability with International Best Practices.

The proposed model aligns well with international best practices in knowledge management and health system optimization. Similar frameworks have been successfully implemented in countries with advanced healthcare systems, resulting in significant improvements in care coordination, patient outcomes, and operational efficiency. These international examples provide a robust foundation, suggesting that the proposed KMM for the SGSSS can achieve similar successes if adapted to the local context and continuously refined based on feedback and evolving needs [129].

Evaluating the feasibility of the proposed KMM indicates high potential for its successful implementation, provided careful planning, phased implementation, sufficient resource allocation, and strong stakeholder engagement are ensured. By addressing the initial complexities and leveraging best practices from other health systems, the SGSSS can effectively implement this model to enhance knowledge management, improve healthcare delivery, and achieve better health outcomes for the population [127,131].

## 4. Discussion

Our strategic proposal enables benefits for the improvement of knowledge flows in governance, surveillance, inspection, and control macroprocesses through the use of the proposed model. The contributions of an adequate KMS to address the knowledge errors that occur in the analyzed macroprocesses include the following:

Given that the objective of the macroprocess of stewardship and regulation is to guarantee the processes and results of the defined strategic plan, as well as the management and control of the activities that are carried out to achieve the objectives of the organization, the unnecessary consumption and/or excessive resources used to address the evident problems fundamentally affect the possibility of achieving this objective, given that the system’s adjustment decisions are made using information on the system’s behavior. Consequently, inconsistencies in the base information do not allow for optimal achievement of the expected results [132,133,134].

In the supervision and control macroprocess, the possibility of guaranteeing consistency in all information that flows in this process not only reduces the unnecessary use of resources for possible corrections but also enables the unification of criteria derived from the available information. For example, providers and insurers could easily harmonize their portfolio reconciliation processes, reducing costs and reaching agreements among parties in a more timely manner.

Suppose that the KMS achieves consistency in the information derived from this process. In this case, it also becomes possible that the decisions derived from the control procedures’ results are correct, thus allowing for the solution of the problems that arise in the SGSSS processes in an optimal manner [135,136].

Likewise, the possibility of guaranteeing the quality of the information reduces the risks of inoperative procedures and system delays [137].

The information managed in this macroprocess is the necessary input for the supervision of the EPS, in terms of authorization and permanence, carried out by the Health Superintendency, considering the need to guarantee compliance with the defined requirements [138,139].

The supervision of health systems is a complex task, often influenced by market dynamics and the emergence of new health risks [140,141] or the risk of capturing them [142]. This is particularly relevant in the context of community supervision, where the intersection between supervision and health is not well-understood at present [143]. The effectiveness of supervision activities in ensuring public health and the protection of consumer rights depends on the correct prioritization of supervision targets [144].

In the current context of supervision in the SCS-SGSSS, one of the most pressing issues is the integrity and reliability of the available data. The proposed KMM aims to address significant problems related to the absence, incompleteness, and errors in the data collected and processed across the system. These data integrity challenges are fundamental obstacles that hinder the provision of accurate summary statistics for specific errors [145].

A thorough evaluation of data integrity within the SGSSS highlighted significant issues related to accuracy and consistency, which may hinder the ability to obtain detailed error statistics.

Given these challenges, it is currently infeasible to provide accurate summary statistics on specific errors within SGSSS processes. Therefore, addressing these data integrity issues is a primary objective of the proposed KMM. Implementing a centralized platform for data capture and management aims to enhance the accuracy and reliability of data, enabling the future generation of detailed statistics [146].

The KMM is expected to tackle these issues through the use of a centralized data repository, Standardized Data Entry Protocols, and Regular Data Audits. These improvements will enhance the quality of data and enable the provision of accurate summary statistics, providing clearer insights into the operational challenges faced within the context of the SGSSS. Overall, addressing the existing data integrity limitations through the KMM is expected to improve both the reporting accuracy and healthcare outcomes in Colombia.

## 5. Conclusions

The proposed model offers a comprehensive approach to knowledge management, considering the complexities of the SSC-SGSSS in Colombia.

The model’s original and flexible design allows it to adapt to the needs of the system, executing several processes either simultaneously or cyclically.

The model leverages national and international references, as well as experiences gleaned from external references and the work team, demonstrating a comparative and inclusive approach to knowledge management.

The model emphasizes the importance of continuous learning and feedback, and its stages can be executed simultaneously to complement each other.

The proposal in this study underscores the importance of comparing various sources—including theoretical and conceptual frameworks—in order to develop a robust and effective knowledge management model.

While the model shows promise, further research is needed to evaluate its effectiveness in practice and to refine it based on feedback and real-world lessons, highlighting the dynamic nature of knowledge management and the need for such models to evolve and adapt over time.

Finally, a desirable aspect to facilitate the development of the KMS in the SC-SGSSS would be to establish governance schemes for the information and actions of the supervisor, in addition to the actions of the regulator. The supervision and control system must be democratized, thus avoiding capture, influence, and interference from the supervisor.

## Figures and Tables

**Table 1:** Abbreviations used in this article.

Abbreviation	Full Term (English/Spanish)
ADRES	Administrator of the Resources of the General System of Social Security in Health/Administradora de los Recursos del Sistema General de Seguridad Social en Salud
CAC	High-Cost Account/Cuenta de Alto Costo
CC	Constitutional Court/Corte Constitucional
CGR	Comptroller General of the Republic/Contraloría General de la República
DANE	National Administrative Department of Statistics/Departamento Administrativo Nacional de Estadísticas
DIAN	National Tax and Customs Directorate/Dirección de Impuestos y Aduanas Nacionales
DFS	Fundamental Right to Health/Derecho Fundamental a la Salud
DFP	Ombudsman’s Office/Defensoría del Pueblo
DNP	National Planning Department/Departamento Nacional de Planeación
EPSs	Health Promotion Entities (Health Insurers)/Entidades Promotoras de Salud (Aseguradoras en Salud)
ESE	State social enterprises (Public Hospitals)/Empresas Sociales del Estado (Hospitales públicos)
IETS	Institute of Health Technology Assessment/Instituto de Evaluación Tecnológica en Salud
INS	National Institute of Health/Instituto Nacional de Salud
INVIMA	National Institute of Drug and Food Surveillance/Instituto Nacional de Vigilancia de Medicamentos y Alimentos
IPS	Institutions Providing Health Services/Instituciones Prestadoras de Servicios de Salud
KMM	Knowledge management model
KMS	Knowledge management system
MIPG	Guide for the implementation of knowledge management and innovation within the framework of the integrated planning and management model/Guía para la implementación de la gestión del conocimientos y la innovación en el marco del modelo integrado de planeación y gestión
Minhacienda	Ministry of Finance and Public Credit/Ministerio de Hacienda y Crédito Público
MinTic	Ministry of Information and Communications Technologies of Colombia/Ministerio de Tecnologías de la Información y las Comunicaciones de Colombia.
MSPS	Ministry of Health and Social Protection/Ministerio de Salud y Protección Social
NHS	National Health System (British)
NUIP	Unique Personal Identification Number/Número Único de Identificación Personal https://www.registraduria.gov.co/Avanza-la-aplicacion-del-Nuip-en-Colombia.html, accessed on 23 July 2020
ONS	National Health Observatory/Observatorio Nacional de Salud
PBS	Health Benefits Plan/Plan de Beneficios en Salud
PGN	Attorney General’s Office/Procuraduría General de la Nación
PIC	Collective Intervention Plan/Plan de Intervenciones Colectivas
PMS	Maximum Health Budgets/Presupuestos Máximos en Salud
RC	Contributory Regime/Régimen Contributivo
RISS	Integrated Health Services Networks/Redes Integradas de Servicios de Salud
RNEC	National Registry of Civil Status/Registraduría Nacional del Estado Civil https://registraduria.gov.co/, accessed on 23 July 2020
RS	Subsidized regime/Régimen Subsidiado
RUAF	Unique Registration of Affiliates/Registro Único de Afiliados
SGCME	Management and Control System of Special Measures/Sistema de Gestión y Control de Medidas Especiales
SGSSS	Colombian General Social Security System in Health/Sistema General de Seguridad Social en Salud
SIC	Superintendence of Industry and Commerce/Superintendencia de Industria y Comercio
SISBEN	Identification System of Potential Beneficiaries of Social Programs/Sistema de Identificación de Potenciales Beneficiarios de Programas Sociales https://www.sisben.gov.co/Paginas/que-es-sisben.html, accessed on 23 July 2020
SISPRO	Integrated Social Protection Information System/Sistema Integrado de Información de la Protección Social https://www.sispro.gov.co/Pages/Home.aspx, accessed on 23 July 2020
SIVIGILA	National Public Health Surveillance System/Sistema Nacional de Vigilancia en Salud Pública https://portalsivigila.ins.gov.co/, accessed on 23 July 2020
SOAT	Mandatory Traffic Accident Insurance/Seguro Obligatorio Accidentes de Tránsito
SSC	Supervision and Control System/Sistema de Supervisión y Control
SSC-SGSSS	Supervision and Control System of the Colombian General Social Security System in Health/Sistema de Supervisión y Control del Sistema de Seguridad Social en Salud
Superfinanciera	Financial Superintendence of Colombia/Superintendencia Financiera de Colombia
Supersalud	National Health Superintendency/Superintendencia Nacional de Salud
Supersociedades	Superintendence of Companies/Superintendencia de Sociedades
SNGR	National Disaster Risk Management System/Sistema Nacional de Gestión del Riesgo de Desastres
UPC	Capitation payment unit/Unidad de Pago por Capitación

Source: Authors’ own elaboration.

**Table 2:** Information errors in governance, stewardship, and regulation macroprocesses in the SGSSS of Colombia.

Category	Description
Duplication of information errors	There is a multiplicity of information sources without a unified design or common structure. Some sources are the Ministry of Health, National Administrative Department of Statistics, National Registry of Civil Status, Ministry of Finance, National Planning Department, National Institute of Health, High-Cost Account, National Institute of Drug and Food Surveillance, Institute of Health Technology Assessment, among others.
	In the absence of a fully unified system, any difference in the contents and/or quality of the same object generated lead the local information systems owned by the different actors to interpret it as belonging to a different actor of the system, not having the capacity/possibility to understand that it is the same record.
Hidden, erroneous, or non-existent information errors	There is no single repository or fully centralized system that either (as a first option) stores the information or (as a second alternative) can ensure the validity, reliability, and consistency of the information in all the instances in which it is used, generating a risk of “hidden information” regarding where the information is stored in the first place. It may exist, but it is not in the domain of the entities that require it and, therefore, does not contribute to improving processes.
	There is no information on the population without access to the system, inequity in access, the conditions of the dominant position of EPS vis-à-vis providers and of these providers vis-à-vis health workers, or even a combination of these shortcomings.
	Situations can be detected in which there is a lack of information on epidemiological risks by EPS that needs to be provided. It is not available and/or cannot be easily consulted by the actors who need it. A lack of information on the sufficiency of supply capacity (physical infrastructure, availability of talent) is also detected (e.g., supplies and medicines).
	In most cases, there is a deficiency in the quantity and quality of information related to the quantity and cost of health benefits, which is accentuated in the subsidized regime.
	There is no concordance of information in the different repositories or databases belonging to some entities, a situation necessitating that there exists an adequate relationship among glosses, collections, accounts receivable, and accounts payable, which are managed by some of the system’s actors.
Existence of unnecessary or excessive consumption of time and/or resources caused by the errors identified	Shortcomings in the integrity of information generate the excessive and unnecessary consumption of resources in the search for solving their consequences.
	Because of regulations and the structure of the current system, hospitals must generate and report a large amount of information arising from their operations, some of which is sometimes unnecessary and is not used in its entirety in decision-making at the different levels that request it (e.g., hospitals, insurers, Territorial Directorates of Health, Superintendence of Health, and Ministry of Health).
	Generation and transmission of information that is optional. In addition to avoiding the inconveniences mentioned above to guarantee the necessary quality levels, it generates reprocesses, misuse of resources, and cost overruns in the provision of services because of the need to generate reports. This situation is accentuated by the fact that there are multiple sources and different recipients of this information.

Source: Authors’ own elaboration.

**Table 3:** Errors due to duplicate information in the SSC-SGSSS of Colombia.

Category	Description
Non-unified source of channeling for the input or generation of information to the same system	Two actors of the SGSSS generate the same information through the use of different sources or the same one at different times. Then, some content of this information may be transformed, complemented, or modified; for example, this situation occurs when the ADRES publishes a monthly publication of affiliates by the EPS simultaneously with the one of the Social Protection Information System (SISPRO, by its acronym in Spanish) as an official source.
	The quarterly report on the quality indicators of the Health Provider Institutions (IPSs) that apply to each of the services provided by Resolution 256 of 2016 (effectiveness, risk management, management experience, and safety) must be submitted to the SISPRO of the Ministry of Health. The IPS must also report to the EPS to consolidate the corresponding information for all of the contracted providers and report to the same system. The project team found that the consolidated information at the grouping of the EPS or Department levels is not always equivalent to the consolidation of the information that the IPS reports individually.
	Some users of the system are registered under one category but, when their situation changes and they are registered in a new one, the previous registration still needs to be deleted. An example is that of live births, which are initially registered in the Single Registry of Affiliates to the SGSSS (RUAF, by its acronym in Spanish) associated with the identity document of the father or mother and with an additional digit that identifies them. With time, the same person is registered with a new identity document assigned by the legislation (NUIP of the Civil Registry of Birth, Identity Card, or Citizenship Card). There have been cases—especially in rural areas—where databases are not cleaned, leading to this type of risk.
Duplication of information originating in this same macroprocess	Any of the actors with access to the databases executes processes of entry, consultation, or modification of certain information in some or multiple of the modules of the different systems used in the SGSSS. Upon completion of its process and after its use or modification, it records these changes in the system as valid information and unique, without having carried out debugging processes.
	Official sources for health management indicators (SIVIGILA, SISPRO; quality indicators) are not purified data, nor do they have prior validation processes, which may affect the results for an EPS.
	In the financial processes of collecting and paying invoices and accounts between the IPS and EPS, cases are evident in which the information endorsed by each of these instances presented differences, which, in some cases, are significant. This situation is generated when there are different documents or invoices for the same situation—a situation that, in some cases, activates conciliation processes.
	Because of the variety of corporate figures existing in Colombian legislation, there are differences in the control entities that apply to some entities and others. For example, in tax matters, the jurisdiction is the National Tax and Customs Directorate (DIAN, by its acronym in Spanish). However, in terms of other financial and accounting aspects, other control entities take this variety into account, such as the Superintendence of Solidarity Economy for Cooperatives or the Superintendency of Family Subsidies for Savings Banks of Family Compensation, requiring the generation of different types of reports for these control entities, which must be sent in different formats and use different media, generating a high risk of duplication or mistakes in the same information.

Source: Authors’ own elaboration.

**Table 4:** Hidden, erroneous, or non-existent information errors in the SSC-SGSSS of Colombia.

Category	Description
Under-registration	The under-registration of live births, especially in rural sectors, is due to social or cultural factors, as the registration of births takes time and may be postponed over time. The greater risk of under-registration of the population affects other databases of different types, causing differences when consulting them in the repositories of the Civil State Registry, the MSPS, and the Notaries, among others.
	The SGSSS needs to improve the management of opportunity and access indicators in the system, where the calculations start from the first attempt of a user to access any of the administrative or medical services contracted. However, instead, the times and costs begin to be counted only from the moment this user gains access to the system in cases such as assigning a medical appointment, delivery of medications, or authorization of a procedure. The opportunity is only counted when the answer is affirmative, and the indicator will always be positive. However, countless cases are evident in which precisely the main complaints that users fear have to do with the lack of opportunity and the great difficulties in accessing a process flow that solves their situation.
Confidentiality	In health risk management, access to this information is restricted in response to patient confidentiality policies and their medical condition. However, some actors and stages of the macroprocess need to use information in a consolidated and anonymized manner and not in a particular way—a situation that would not violate these confidentiality policies.
Different sources	In the creation and/or calculation of health indicators that require information from different sources, some of the sources may present information that needs to be more consistent and verified, affecting the result of the calculation and, thus, the indicator would present the same flaw itself. In subsequent information that uses the associated results as input, under-recording or registration exceeding the real case may occur.
Low quality	Multiple sources cause errors when calculating the Capitation Payment Unit and Maximum Budgets in the Subsidized Regime because of errors and/or low quality of this information when the data source is a public provider. In these cases, the reliability levels for different calculations and activities deviate from the acceptable standards in the system.
Delays in procedures	There are multiple errors in the management of complaints. They are reported as solved by sending generic responses that do not offer an effective solution to user cases when, in reality, they have not been resolved, or when response times are recorded from the last communication from the user before achieving the required solution, but previous contact attempts (even from months ago) are ignored.
Wrong reports	Aspects such as response times, assignment of appointments, delivery of medications, and the information reported to SISPRO are not totally correct, leading to positive indicators for the EPS and IPS that need to be adjusted to reality, which prevent decision-making corrective measures to improve the SGSSS.
Delayed information	There is information that is only formalized or accounted for in periods other than those in which it is generated, such as that related to the generation and payment of invoices or indicators of delay in care. Using a certain mechanism, the registration of this information is formalized in periods after its occurrence, ensuring that the results of some of these indicators do not affect the evaluation of any of the actors. In accounting matters, this practice is especially evident in the last and first months of each year, a situation that provides favorable results for a specific actor.
Untimely reports	The untimely reporting of some information that different actors must send to the control entities, despite the regulations clearly defining the times within which these reports must be made, is a typical case of non-existent information that needs to be generated. In this case, this is due to procedural errors and also to an omission in any of the stages of the macroprocess itself or of any of those that feed it.

Source: Authors’ own elaboration.

**Table 5:** Components of the proposed model for the SSC-SGSSS of Colombia.

Component	Description
Macroprocesses	These are the basic units of analysis, which represent functions and logic and interact with other macroprocesses, thus sharing knowledge flows. Five macroprocesses of the SGSSS are identified in the model as follows: (i) governance; (ii) affiliation; (iii) financing; (iv) risk management; and (v) surveillance, inspection, and control.
Knowledge management strategy	This defines the organization’s objectives and goals concerning the effective use of knowledge and establishes the direction and approaches that will be followed to capture, store, organize, distribute, and apply knowledge.
Organizational culture	This is a crucial factor that determines the success of knowledge management. A culture that values and encourages knowledge sharing, continuous learning, and collaboration can be expected to facilitate the successful implementation of a knowledge management model.
Knowledge management subprocesses	These are the specific activities and practices for knowledge management in the organization. Some examples are the creation of communities of practice, the documentation of best practices, the management of lessons learned, and knowledge transfer.
Information technology	These are tools and technological systems that facilitate knowledge management, which can include databases, content management systems, collaboration platforms, intranets, and other applications that allow the efficient capture, storage, and access of knowledge.
Human resources	These people identify, share, and effectively apply knowledge. It is necessary to have trained and motivated personnel who can perform clear roles and responsibilities related to knowledge management.
Technology and transactional databases	These are the means that allow for the storage of large volumes of data in a structured way, carrying out transactions that guarantee the integrity of the data, accessing and modifying the information concurrently and securely, and searching and retrieving relevant information.
Measurement and evaluation	These are the indicators and metrics used to evaluate the effectiveness of knowledge management, which can include tracking the use of the knowledge base, participation in communities of practice, user satisfaction, and its impact on organizational outcomes.
Organizational learning	This is the ability to learn from experience, adapt to changes, and continuously improve knowledge management processes and practices. A knowledge management model must be connected with the organization’s continuous learning cycle

Source: Authors’ own elaboration.

**Table 6:** Relationship between traditional models and the innovative proposal of a knowledge management model for the Colombian SSC-SGSSS.

Proposed Model	Stages of Traditional Models	Objective
PHASE 1. Initial diagnosis	Enlistment	Identify knowledge needs and gaps to guide the design and implementation of the knowledge management model.
PHASE 2. Strategic proposal for the improvement of knowledge flows	Knowledge capture and gathering	Design actions, flows, and components that ensure the adequate input and flow of information in the macroprocesses of the knowledge management model.
	Knowledge management, distribution, and application	
PHASE 3. Follow-up and analysis	Knowledge re-use or recycling	Evaluate the use of knowledge, analyze the results, and provide feedback to improve the effectiveness and impact of the knowledge management model.
	Knowledge permanence and use	

Source: Own elaboration.

## Data Availability

The original contributions presented in this study are included in this article/Supplementary Materials. Further inquiries can be directed to the corresponding author.

## Supplementary Materials

The following supporting information can be downloaded at: https://www.mdpi.com/article/10.3390/jmahp12030019/s1: Supplementary Materials S1–S5. Detailed results of the findings for all of the analyzed macroprocesses.
