# Supplementary material for: Proposal of a Knowledge Management Model for Complex Systems: Case of the Supervision and Control Subsystem of the Colombian Health System
Source: J Mark Access Health Policy. 2024 Aug 21;12(3):224–51. doi: 10.3390/jmahp12030019 (PMC11348183; doi:10.3390/jmahp12030019)
Supplement: Supplementary file 1 [file jmahp-12-00019-s001.zip › S1 Governance Stewardship Regulation Macroprocesses Description.pdf]

Macroproceso 1. Gobernanza / Rectoría / Regulación

1. Objetivo.

   El objetivo del Macroproceso de Rectoría y Regulación es el de dirigir, orientar y coordinar el SGSSS para garantizar a toda la población el acceso a los servicios de salud de manera oportuna, eficaz y con calidad para la preservación, el mejoramiento y la promoción de la salud.

   El Macroproceso 1 busca garantizar el derecho fundamental a la salud mediante la adopción de políticas para asegurar la equidad de trato y oportunidades en el acceso a las actividades de promoción, prevención, diagnóstico, tratamiento, rehabilitación y paliación para todas las personas.

   De acuerdo con la meta 3.8 de los Objetivos de Desarrollo Sostenible -ODS de la OMS y de la OPS, relacionada con la obtención de la cobertura universal en salud, el Sistema innovador de gestión del conocimiento propuesto en este trabajo se alinea con esta meta y propone que el objetivo del "Macroproceso 1. Rectoría y Regulación" sea monitorear, medir, dar seguimiento, controlar y gestionar el presente y el futuro de las entidades involucradas en el SGSSS, logrando de esta forma establecer los lineamientos para el funcionamiento del SGSSS y las funciones de cada actor dentro de éste, así como los mecanismos de seguimiento el control para lograr los objetivos del mismo.

   Como marco normativo dentro del cual se desarrolla este Macroproceso hace referencia a la Ley 100 de 1993 - Artículo 152, la Ley 1122 de 2007, Ley 1438 de 2011 y la Ley 1751 de 2015.

2. Conocimiento inicial que alimenta el Macroproceso.

   En el Macroproceso 1 referente a la Rectoría del sistema y complementando su definición como el componente inicial para este sistema de gestión del conocimiento, sirve como proceso de intermediación y de entrada para los insumos de otros sistemas del entorno, necesarios para funcionamiento y gestión.

   En ese orden de ideas, esta información se clasificará de acuerdo al origen de la misma, definiéndose entonces las categorías referentes a la información proveniente de los usuarios del sistema (U), a la información proveniente de otros actores o componentes del SGSSS (C) y a la información proveniente del entorno (E).

   Como información complementaria para el Macroproceso, existen comisiones asesoras sobre diferentes aspectos del Sistema, donde tiene participación la sociedad civil.

   El inventario inicial de esta información previa o "información de entrada" en la categoría definida para otros componentes del sistema (C) incluye la normatividad e información originada o tramitada por el Ministerio de Salud y Protección Social (Minsalud) y por la Superintendencia Nacional de Salud (Supersalud), información del Instituto Nacional de Salud (INS), información de las Entidades Promotoras de Salud (EPS), información de las Instituciones Prestadoras de Servicios de Salud (IPS) y de otros prestadores, información de los entes territoriales, entre otros. La información asociada a esta categoría incluye también los Registros Individuales de Prestación de Servicios -RIPS y las diferentes leyes, decretos y resoluciones surgidas de alguna de estas instancias, información asociada al riesgo financiero de las EPS, asociadas al riesgo para los usuarios, asociada al posible uso ilegal de los recursos y dentro del proceso de Peticiones, Quejas, Reclamos y Denuncias –PQRD.

   En cuanto a la información entrante correspondiente a la categoría relacionada con el entorno (E) se destaca información originada en instancias y entidades tales como la

Defensoría del Pueblo, la Contraloría General de la República, la Registraduría Nacional del Estado Civil, el Departamento Administrativo Nacional de Estadística DANE, así como una gran variedad de investigaciones surgidas desde la Academia. Dentro de esta categoría y asociada a las instancias anteriores se identifican diferentes tipos de información financiera y técnico-científica, información tecnológica, indicadores sociodemográficos y económicos a nivel local, departamental y nacional.

El procesamiento y trámite de la información surgida y de la que se hace uso en las categorías anteriormente descritas, encuentra su utilidad para este Macroproceso en aspectos tales como al permitir conocer y aplicar las normas que deben aplicar los diferentes componentes del SGSSS en casos específicos, que incluye la normatividad que debe ser atendida por las EPS, los prestadores de servicios de salud, proveedores de tecnologías en salud y los usuarios del sistema, así como la toma de decisiones adecuadas e informadas para garantizar el acceso a los servicios de salud para la población asegurada.

La información generada como entrada al Macroproceso de acuerdo con lo explicado anteriormente, evoluciona y se transforma teniendo como resultado que la misma haya sido complementada con nueva información o se transforme, alimentando de esta manera etapas tanto del mismo Macroproceso como de LOS otros Macroprocesos definidos y que pueden activarse simultáneamente o de manera posterior. Esta evolución permite que el objetivo definido en esta instancia se complemente, identificándose la necesidad de que las actividades de rectoría y regulación permitan analizar la información disponible para definir nuevas normas y lineamientos para el sector, activándose un proceso de retroalimentación y generación de nuevo conocimiento que propende por la mejora continua del Macroproceso. Esta evolución permite la generación de nueva información sobre gestión del riesgo y sobre indicadores de cumplimiento en la gestión de las EPS, en un proceso dinámico que realimenta al sistema permitiendo su evolución.

Como parte de la búsqueda del cumplimiento de los objetivos definidos, en este Macroproceso también se debe garantizar la identificación específica de la normatividad del Minsalud que rigen el funcionamiento y aplicación de estas normas por parte de la Supersalud.

A continuación, se detallan los principales aspectos contenidos en los artículos 156 y 173 de la ley 100 de 1993 los cuales han sido definidos como precursores del conocimiento inicial que alimenta el Macroproceso 1.

Ley 100 de 1993, Artículo 156, literal a: El Gobierno Nacional dirigirá, orientará, regulará, controlará y vigilará el servicio público esencial de salud que constituye el Sistema General de Seguridad Social en Salud

Ley 100 de 1993, Artículo 173: de las funciones del ministerio de salud.

Son funciones del Ministerio de Salud además de las consagradas en las disposiciones legales vigentes, especialmente en la Ley 10 de 1990, el Decreto ley 2164 de 1992 y la Ley 60 de 1993, las siguientes:

a) Formular y adoptar, en coordinación con el Ministerio de Trabajo y Seguridad Social, las políticas, estrategias, programas y proyectos para el Sistema General de Seguridad Social en Salud, de acuerdo con los planes y programas de desarrollo económico, social y ambiental que apruebe el Congreso de la República.

b) Dictar las normas científicas que regulan la calidad de los servicios y el control de los factores de riesgo, que son de obligatorio cumplimiento por todas las Entidades Promotoras de Salud y por las Instituciones Prestadoras de Servicios

de Salud del Sistema General de Seguridad Social en Salud y por las direcciones seccionales, distritales y locales de salud.

c) Expedir las normas administrativas de obligatorio cumplimiento para las Entidades Promotoras de Salud, por las Instituciones Prestadoras de Servicios de Salud del Sistema General de Seguridad Social en Salud y por las direcciones seccionales, distritales y locales de salud.

d) Formular y aplicar los criterios de evaluación de la eficiencia en la gestión de las Entidades Promotoras de Salud y por las Instituciones Prestadoras de Servicios de Salud del Sistema General de Seguridad Social en Salud y por las direcciones seccionales, distritales y locales de salud.

e) Ejercer la adecuada supervisión, vigilancia y control de todas las entidades comprendidas en los literales b) a h) del artículo 181 de la presente ley y de las direcciones seccional, distrital y local de salud, excepto la Superintendencia Nacional de Salud.

f) El Ministerio de Salud reglamentará la recolección, transferencia y difusión de la información en el subsistema al que concurren obligatoriamente todos los integrantes del sistema de seguridad social de salud independientemente de su naturaleza jurídica sin perjuicio de las normas legales que regulan la reserva y exhibición de los libros de comercio. La inobservancia de este reglamento será sancionada hasta con la revocatoria de las autorizaciones de funcionamiento.

3. Síntesis de la descripción procedimental.

Este Macroproceso se relaciona directamente con la política regulatoria del Estado en lo concerniente al SGSSS.

Con base en la información y los reportes epidemiológicos, en las políticas públicas, en las peticiones de los usuarios, en las peticiones de los gremios y de los diferentes actores del sistema, por reportes del INVIMA y por reportes del INS entre otras fuentes, el Minsalud define planes de acción en respuesta a posibles riesgos de salud para la sociedad tomando como base diferentes criterios de política económica y de salud pública definidos por el Estado.

Cuando estos análisis elaborados con criterios técnicos y científicos advierten de una posible anomalía en estos niveles de riesgo, específicamente en el aseguramiento en salud, es posible que se active una de dos posibles regulaciones:

La primera tiene que ver con los requisitos de habilitación y de permanencia financiera de las entidades, que Supersalud inspecciona y vigila permanentemente y cuyo resultado deriva en la normatividad que se renueva en cuanto a estos requerimientos, asociada a nuevos Decretos o Resoluciones, reglamentados por el Ministerio de Salud y Protección Social. Esta situación activa también los procedimientos de vigilancia y control que se ajustan con la participación de los actores definidos para tal fin.

La segunda tiene que ver con la regulación de los precios del mercado, donde el valor unitario que reciben las aseguradoras como prima se denomina Unidad de Pago por Capitación (UPC). Este valor es establecido anualmente por el Minsalud en cabeza de la Dirección de Beneficios, Costos y Tarifas como resultado de un análisis técnico definido para tal fin.

Este análisis se basa en la información consignada en la base de suficiencia para la UPC. Esta base contiene la totalidad de información de servicios prestados anualmente por la totalidad de actores de la red, donde la consolidación de los datos registrados por todos los prestadores es reportada por la aseguradora a la que pertenece.

Como medio de validación, esta base reportada anualmente es analizada bajo criterios de consistencia donde se depura y se asegura su confiabilidad. Con esta se establece el valor anual de la denominada canasta de beneficios la cual es formalizada de manera simultánea con la información del valor de la UPC.

La definición de este plan de beneficios se hace con base en los Códigos Únicos de Procedimientos en Salud CUPS, con base en los Códigos Únicos de Medicamentos en Salud CUMS y con base en los diagnósticos.

Esta definición procedimental también considera unas condiciones de habilitación y permanencia para los prestadores, las cuales deben ser cumplidas para poder ser contratados por las EPS.

Así mismo, cuando el Instituto Nacional de Vigilancia de Medicamentos y Alimentos (INVIMA) otorga los registros sanitarios para las tecnologías y para los medicamentos está dando el aval para que los mismos sean ofertados dentro del Sistema por parte de la red que lo conforma, al tratarse de la entidad debidamente autorizada para emitir este tipo de avales.

4. Errores de duplicidad de información.

   El análisis hecho por el equipo de trabajo de esta consultoría permite identificar que existe una multiplicidad de fuentes de información que se involucran en el sistema y que no tienen un diseño unificado ni una estructura común, situación que genera un alto riesgo de duplicidad, inconsistencias y de errores en la información. Entre las entidades que originan esta información o la transforman y de acuerdo con la descripción presentada en este Macroproceso se encuentran el Minsalud, DANE, la Registraduría Nacional, el Minhacienda, el DNP, el INS, la Cuenta de Alto Costo, el (Invima), el Instituto de Evaluación Tecnológica en Salud (IETS) y las diferentes entidades territoriales que se involucran en el proceso.

   En términos generales, existe un alto riesgo de duplicidad porque hay algunas normas que contradicen lineamientos tenidos en cuenta en otras normas o que plantean aspectos de manera parcial y hacen necesario la creación de nueva información o la expedición de nuevas normas para complementar.

   Existe también riesgo de duplicidad parcial y posibles inconsistencias en la misma, en información contenida en algunos reportes producidos o emitidos por organismos diferentes, pero que no siempre son coincidentes entre sí.

   Estos riesgos o la materialización de duplicidad parcial o total conllevan a que en el sistema exista información duplicada en su totalidad que ocasiona desperdicio de recursos, o duplicada parcialmente, pero con algunas diferencias lo que ocasiona inconsistencias.

   Las afectaciones causadas por esta situación redundan en riesgos para la toma de decisiones a nivel país al no existir unicidad en los indicadores de calidad reportados por y hacia las EPS y por / hacia las IPS.

   Al nivel de la información contenida en los diferentes reportes que se generan en la ejecución de los Macroprocesos, también existen riesgos de duplicidad de duplicidad o de inconsistencias parciales o totales para un mismo individuo, cuando existen diferentes actores del sistema que recaudan la misma información, en diferentes momentos o circunstancias. Al no existir un sistema totalmente unificado, cualquier diferencia en los contenidos y/o en la calidad de los mismos, genera que los sistemas de información locales que poseen los diferentes actores interpreten la misma como perteneciente a un actor diferente del sistema, no teniendo la capacidad/posibilidad de entender que se trata del mismo registro.

Todo lo anteriormente evidenciado genera altos riesgos en los procesos de identificación, análisis, interpretación y toma de decisiones en las diferentes instancias y en los diferentes Macroprocesos del sistema.

5. Errores de información oculta, equivocada o inexistente.

   Adicionalmente a lo anteriormente evidenciado con los riesgos de duplicidad de información, el sistema también presenta riesgos de información con errores en su contenido o ausencia de estos, lo que redunda en que el sistema corra el riesgo de contener información equivocada, incompleta o inexistente.

   Adicionalmente y dado que no existe un repositorio único ni un sistema totalmente centralizado que, o como primera opción almacene la información o como segunda alternativa pueda asegurar la validez, fiabilidad y consistencia de esta en todas las instancias en las que se utilice, se genera un riesgo de "información oculta" donde la misma puede existir, pero no es del dominio de las entidades que la requieren y por ello no aporta a la mejora de los procesos.

   Es importante anotar que lo anterior redunda en que, en algunas ocasiones, la información necesaria para la toma de decisiones presenta niveles de calidad que adolecen de los problemas explicitados en los párrafos anteriores y por tanto, la oportunidad de la información que alimenta este Macroproceso no es la adecuada en la totalidad de las ocasiones en las que se la requiere. Esta situación genera problemas en el funcionamiento del sistema dado que se necesita información de calidad para que este Macroproceso tome las mejores decisiones para el sistema.

   En algunos otros casos, no existe información de población sin acceso al sistema, o sobre inequidad en el acceso, o sobre condiciones de posición dominante de EPS frente a prestadores y de estos prestadores frente a trabajadores de la salud, o incluso, una combinación de estas falencias. En ese sentido pueden detectarse situaciones en las que falta información sobre riesgos epidemiológicos por EPS y sobre la zona geográfica de incidencia, o esta no se encuentra disponible y/o no es de fácil consulta para los actores que la necesitan. También se detectó falta de información sobre la suficiencia de la capacidad de oferta (infraestructura física, disponibilidad de talento humano e insumos y medicamentos).

   Es importante anotar que la mayoría de estos casos donde se presenta deficiencia en la cantidad y en la calidad de la información relacionada con la cantidad y costo de las prestaciones de salud, la cual se acentúa en el régimen subsidiado.

   Como parte de este diagnóstico también se pudo evidenciar que en diferentes situaciones no existe concordancia de información en los diferentes repositorios o bases de datos pertenecientes a algunas entidades, situación necesaria para que exista una relación adecuada entre las glosas, los recobros, las cuentas por cobrar y las cuentas por pagar, las cuales son gestionadas entre algunos de los actores del Sistema.

   Lo anteriormente descrito evidencia que tanto los problemas generados por la información duplicada, ya sea total o parcialmente, como los problemas por información equivocada, oculta o inexistente, afectan en gran medida el Macroproceso de regulación y rectoría, dado que estas falencias afectan el seguimiento adecuado en el tiempo y genera posibles retrasos en la toma de decisiones, dificultando un análisis integral sobre los posibles efectos de los cambios normativos.

6. Existencia de un consumo innecesario o excesivo de tiempo y/o recursos, ocasionado por los errores identificados.

   La frase con la que culmina el apartado anterior permite aclarar que estas falencias en la integridad de la información generan un consumo excesivo e innecesario de

recursos en la búsqueda de solucionar las consecuencias de estas.

Por ilustrar uno de estos consumos excesivos e innecesarios, se puede nombrar el caso de las IPS, las cuales por normatividad y estructura del sistema actual deben generar y reportar una gran cantidad de información surgida de su operación, pero una porción de la misma en algunas ocasiones es innecesaria y la misma no es utilizada en su totalidad en la toma de decisiones en los diferentes niveles que los solicitan (IPS, EPS, DTS, Supersalud, Minsalud, entre otros).

Esta generación y transmisión de información que no es necesaria, además de sortear los inconvenientes enunciados anteriormente para tratar de garantizar los niveles de calidad necesarios, genera reprocesos, mal uso de recursos y sobrecostos en la prestación de servicios por la necesidad de generar reportes. Esta situación se ve acentuada con el hecho de que existen múltiples fuentes y diferentes destinatarios de esta información, acentuando lo descrito.

7. Contribución de la solución de estos errores al correcto funcionamiento del modelo de gestión del conocimiento.

   Dado que el objetivo del Macroproceso de rectoría y regulación consiste en garantizar los procesos y resultados de la planeación estratégica que se ha definido, así como la gestión y control de las actividades que se adelantan para obtener los objetivos de la organización, estos consumos innecesarios y/o excesivos de recursos utilizados para aminorar los problemas evidenciados, afectan de manera fundamental la posibilidad de consecución de este objetivo dado que con la información del comportamiento del sistema es que se toman las decisiones de ajustes del mismo. Y como consecuencia, las inconsistencias en la información de base no permiten el logro óptimo de los resultados esperados.
