# Supplementary material for: Proposal of a Knowledge Management Model for Complex Systems: Case of the Supervision and Control Subsystem of the Colombian Health System
Source: J Mark Access Health Policy. 2024 Aug 21;12(3):224–51. doi: 10.3390/jmahp12030019 (PMC11348183; doi:10.3390/jmahp12030019)
Supplement: Supplementary file 1 [file jmahp-12-00019-s001.zip › S2 Affiliation Macroprocess Description.pdf]

Macroproceso 2. Afiliación

1. Objetivo.

   Al considerarse como el segundo Macroproceso del SGSSS de Colombia de acuerdo a la propuesta de este documento y a que tiene una alta carga operativa y por tanto de uso de la información generada por el entorno y por otros Macroprocesos, se ha definido como objetivo principal de la afiliación el garantizar el acceso a los servicios de salud a la totalidad de la población, según lo establecido en la ley Estatutaria, garantizando la universalidad del aseguramiento y el acceso a estos servicios.

   El Macroproceso de afiliación permite conocer, de acuerdo con la información suministrada de afiliados, el régimen al cual van a ser objeto de afiliación, para la prestación de los servicios de salud.

   Esta caracterización permite la validación de los derechos y obligaciones del afiliado y su pertenencia al sistema, permitiendo el dinamismo y la continuidad del proceso para garantizar su actualización permanente.

2. Conocimiento inicial que alimenta el Macroproceso.

   La información que alimenta esta sección tiene que ver entre otra con información de afiliados a las EPS, información de las EPS como tal e información del SISBEN, proveniente de los formularios y canales definidos para tal fin, conteniendo información financiera y sociodemográfica de la población.

   Durante el Macroproceso, esta información permite surtir el proceso de compensación de recursos de la UPC y se transforma porque cambian los afiliados entre régimen, los valores de las cotizaciones cuando aplica y por lo tanto los ingresos al sistema.

   Atendiendo a la definición del Macroproceso de afiliación y a la información que se tramita como parte de su gestión, esta información se clasificará de acuerdo con el origen de la misma, definiéndose entonces las categorías referentes a la información proveniente de los usuarios del sistema (U), a la información proveniente de los usuarios (U), de otros actores o componentes del SGSSS (C) y a la información proveniente del entorno (E).

   Como se mencionó previamente, este Macroproceso marca el inicio de la operación mediante la vinculación de usuarios. Este proceso resulta en la generación del contenido de las bases de datos, las cuales se alimentan a través del Formulario Único de Afiliación, Registro y Novedades al SGSSS.

   La información consignada en este formulario se transfiere al sistema de gestión del conocimiento, específicamente en la categoría üsuarios (U)", ya sea durante el momento de afiliación o cuando se produce algún cambio en los registros de un usuario en particular.

   En los casos en los cuales la normatividad así lo defina para un grupo de afiliados en particular, el instrumento de recolección se denomina "Formularios de afiliación y encuesta SISBEN".

   Dentro de la categoría de otros actores o componentes del SGSSS (C) puede agruparse la información relevante y útil para el sistema de los actores que intervienen en el proceso de afiliación y que serán detallados en este capítulo, dentro de la cual se puede destacar la información Financiera, la Técnico-Científica y la información Tecnológica asociada a estos organismos.

   El Macroproceso 2 inicia con una solicitud de afiliación por parte de un ciudadano utilizando los diferentes canales que el SGSSS tiene definidos (formularios, SAT) o con el reporte de novedades sobre aspectos sociodemográficos del afiliado. Asimismo,

con el fin de mejorar la cobertura del sistema, a través del decreto 064 de 2020 se definió la afiliación de oficio como instrumento para la afiliación en una EPS, de aquella población que se encuentre sin aseguramiento en salud por parte del Sistema General de Seguridad Social en Salud - SGSSS, o que se encuentre con la novedad de terminación de la inscripción en una EPS.

Los procedimientos establecidos de acuerdo con la condición de cada persona y su relación con cada uno de los regímenes existentes (contributivo, subsidiado, exceptuado y especiales), se encuentran en la normatividad vigente, Ley 100 de 1993, Ley 691 de 2001, Ley 1122 de 2007, Ley 1438 de 2011, Ley 1955 de 2019 y reglamentados en el Decreto 1953 de 2014, Decreto 780 de 2016, Decreto 064 de 2020, Decreto 616 de 2022).

La solicitud del ciudadano y de acuerdo con esta normatividad, permite que la EPS incluya esta información en su base de datos para formalizar la afiliación, lo que permite que esta sea notificada a las diferentes bases de datos del Sistema.

La pertinencia, calidad y oportunidad de todos los detalles consignados en las bases de datos utilizando alguno de los canales enunciados, permite la identificación plena y actualizada de los afiliados al SGSSS según régimen y ubicación geográfica.

Dentro de los detalles de la información de cada usuario y que alimenta el sistema en la medida en que se adelanta el Macroproceso o en respuesta a algún cambio en las condiciones reportadas inicialmente, tiene que ver entre otros, con aspectos relacionados con la situación sociodemográfica y el estado civil, o con novedades asociadas a nacimientos, fallecimientos, conformación grupo familiar, cambios en el estado civil o cambios en la situación laboral, pertenencia a una categoría étnica o Información sobre alguna condición de discapacidad.

Atendiendo a cada uno de estos cambios de manera particular, se destaca que los mismos pueden ser transitorios o permanentes.

La información previamente descrita que alimenta las bases de datos del sistema en un primer momento y que de acuerdo con su evolución genera nueva información, también se convierte en información que alimenta este mismo Macroproceso o forma parte de información inicial para otros Macroprocesos.

De manera inicial se puede destacar que en este caso es útil al momento de proyectar los ingresos anuales esperados en el sistema por cotizaciones y las necesidades de recursos del Estado para cubrir costos del Régimen Subsidiado.

3. Síntesis de la descripción procedimental.

   El Macroproceso de afiliación tiene un componente de caracterización de la población para especificar la información sociodemográfica para los nuevos afiliados.

   Para el segmento de la población que no tiene capacidad de pago y por tanto pertenecerá al Régimen Subsidiado, esta caracterización se realiza mediante encuestas que sirven como instrumento para clasificar a cada persona en una categoría de SISBEN.

   Para el segmento de la población con mayor capacidad de pago que tiene un contrato laboral o se desempeña como independiente formal, realiza un proceso de afiliación al Sistema el cual está en cabeza de las EPS. La normatividad al respecto describe los trámites y requisitos asociados, los cuales pueden ser consultados para mayor ilustración.

   Las novedades en cuanto a estas características pueden ser registradas en el sistema por medio del aplicativo SAT.

   Dentro de estas novedades y además de los cambios en la información sociodemográfica también se incluyen los cambios en las condiciones para pertenecer a un

régimen, las cuales pueden estar asociadas a los cambios en la empleabilidad o en el tipo de actividad en la que se desempeña un usuario o sus beneficiarios, condición que puede variar también en función de ñas condiciones de pertenencia a alguno de estos regímenes.

La dinámica del empleo en Colombia deriva en una dinámica en la cual estos cambios ocurren con relativa frecuencia, por lo que el sistema contempla también las variaciones en las cotizaciones asociadas. Esta situación deriva en que la ADRES reciba aportes en este sentido ya sea como consecuencia de los trabajadores dependientes, de los independientes formales o por diferentes mecanismos existentes para el caso del régimen subsidiado.

Atendiendo a estas particularidades, existe un flujo de información permanente e importante entre la ADRES y las EPS respectivas que registran la información y los cambios en la misma tanto para los usuarios como para sus beneficiarios que modifica las condiciones sociodemográficas y en tal caso si así se ha definido, las condiciones en las características de la afiliación.

Todos los procesos y la evolución de este Macroproceso generan y tramitan información y conocimiento, situación que exige la existencia de un sistema de gestión flexible.

4. Errores de duplicidad de información.

   El análisis hecho por el equipo de trabajo de esta consultoría permite identificar que existe una multiplicidad de repositorios o bases de datos donde se almacena información del Macroproceso y que son generadas por diferentes instancias del Sistema, situación que origina riesgos de duplicidad de información para un mismo usuario, al estar registrado en bases independientes que no se encuentran integradas entre sí.

   Esta situación es conocida como "multiafiliación" y si bien ha disminuido su ocurrencia en la afiliación al SGSSS, aún se presenta entre la afiliación al SGSSS y a los regímenes de excepción en salud. Ésta debe evitarse mediante el diseño de sistemas de información robustos y que, a su vez permitan el acceso de diferentes componentes del sistema de acuerdo al papel que cumplan en el proceso, situación que también define la posibilidad de gestionar o corregir esta información.

5. Errores de información oculta, equivocada o inexistente.

   Adicionalmente al riesgo de duplicidad de información, el Macroproceso de Afiliación también presenta riesgos de información oculta o inexistente en diferentes casos generados por los mismos usuarios o por algún otro componente del sistema.

   Los casos más notorios de esta ausencia en la calidad de la información tienen que ver en primera instancia con casos en los que el afiliado que hace evasión o elusión al sistema lo cual es responsabilidad de los mismos o en otros casos en que existen reportes tardíos de fallecidos, falta de registro de nacimientos, ausencia de reporte de novedades (movilidad, cambio de documento de identidad, entre otros), subregistro de condiciones especiales de la población en el momento de la afiliación o registro apócrifo de fallecimientos, situaciones atribuibles a otros componentes del Sistema.

6. Existencia de un consumo innecesario o excesivo de tiempo y/o recursos, ocasionado por los errores identificados.

   El análisis hecho por el equipo de trabajo de esta Consultoría permitió evidenciar que existe un consumo innecesario y excesivo de recursos y de tiempo, generado por los reprocesos en la normalización de información registrada en la ADRES por medio de auditoría retrospectivas por afiliados a las EPS y por la gestión para el recobro de

estos recursos por las EPS a las IPS con acuerdo de voluntades con modalidades de pago cápita.

7. Contribución de la solución de estos errores al correcto funcionamiento del modelo de gestión del conocimiento.

   Los errores señalados en los dos apartados anteriores generan inconvenientes que requieren el uso excesivo de recursos del Sistema para solucionarlos, lo que redunda en una disminución en la efectividad de este.

   Las afectaciones causadas por estos consumos innecesarios de recursos afectan el normal funcionamiento, tanto de este Macroproceso como de aquellos que dependen de la información que este aporta al sistema. Dentro de las situaciones inadecuadas que se pueden identificar, están:

   Necesidad de corrección de diferentes tipologías de errores de información, la cual supone el uso de recursos adicionales para lograr a identificar plenamente la pertenencia del afiliado a cada régimen y de esa forma garantizar el cumplimiento de derechos y obligaciones.

   Necesidad de trámites no contemplados, adiciones presupuestales u otro tipo de soluciones surgidas de la afectación de los flujos de caja presupuestados en actores del Sistema tales como las EPS, las IPS, otros Prestadores y la ADRES, entre otras.

   La toma de decisiones erróneas con base en información equivocada perteneciente u originada en este Macroproceso, ocasiona afectaciones en la imagen de la EPS involucrada, situación que genera desconfianza en los usuarios. Este resultado se considera como no adecuado tanto para la institución como para el Sistema, lo que hace necesario la utilización de recursos adicionales tanto para solucionar cada uno de los problemas, como para lograr aminorar el impacto en esta imagen.

   Existe la posibilidad de que haya un desbalance en la relación entre los afiliados al régimen contributivo y subsidiado debido a fallas en el control del cumplimiento de requisitos exigidos para pertenecer al régimen subsidiado.

   El supuesto macroeconómico del sistema partía de que existiría un número superior de afiliados al régimen contributivo que ayudarían a soportar solidariamente la afiliación al subsidiado. Esta situación se ha dado en la mayor parte del tiempo de existencia del sistema, generando presiones sobre el Presupuesto General de la República quien suple la obligación legal de lograr cobertura universal de estos dos regímenes.
