# Supplementary material for: Proposal of a Knowledge Management Model for Complex Systems: Case of the Supervision and Control Subsystem of the Colombian Health System
Source: J Mark Access Health Policy. 2024 Aug 21;12(3):224–51. doi: 10.3390/jmahp12030019 (PMC11348183; doi:10.3390/jmahp12030019)
Supplement: Supplementary file 1 [file jmahp-12-00019-s001.zip › S3 Finance Macroprocess Description.pdf]

Macroproceso 3. Financiación

1. Objetivo.

   El objetivo del Macroproceso de Financiación es definir y asegurar los recursos necesarios para garantizar el goce efectivo del derecho a la salud.

2. Conocimiento inicial que alimenta el Macroproceso.

   En términos generales la mayoría de la información que ingresa a este Macroproceso tiene que ver con las siguientes categorías:

   - Información censal emitida por el DANE y los Entes territoriales
   - Presupuesto General de la Nación asignado al sector salud
   - Sistema General de Participaciones
   - En el RUAF reposa todo el historial de la seguridad social de la población.
   - La información del salario de los trabajadores pertenecientes al Régimen Contributivo y los aportes parafiscales asociados a la cotización de los empleados es registrada en la Planilla Integrada de Liquidación de Aportes (PILA) para establecer el valor de las cuotas moderadoras y los copagos.
   - La información de los ciudadanos pertenecientes al Régimen subsidiado es registrada en el Sistema de Identificación y Clasificación de Potenciales Beneficiarios para Programas Sociales (SISBEN) y es utilizado para determinar el valor de los copagos, la elegibilidad y el nivel de necesidad de los ciudadanos en relación con los programas sociales.
   - La ADRES consolida esta información y distribuye los recursos nacionales y territoriales entre las EPS de régimen contributivo y subsidiado.

   La información anteriormente detallada es esencial para el funcionamiento del Macroproceso, dado que la correcta gestión del conocimiento permite que se garanticen los recursos del SGSSS para así mismo garantizar la prestación de los servicios de salud.

   Por otro lado y en el caso de los recobros, del procesamiento de información a través del MIPRES y del funcionamiento de los procedimientos asociados a los presupuestos máximos, este Macroproceso permite asegurar que los proveedores reciban las contraprestaciones asociadas a su funcionamiento y de esta manera el funcionamiento del Sistema para que sus usuarios puedan hacer uso del mismo y garantizar su derecho a la salud.

   El Macroproceso no tiene dentro de su alcance la modificación de la información de entrada, pero si la transforma al categorizarla y organizarla de acuerdo con la estructura definida para el Sistema, permitiendo la generación de nuevo conocimiento asegurando su actualización permanente y la transmisión de conocimiento hacia otros Macroprocesos.

   De manera complementaria, el Macroproceso de Financiación permite el pago desde la ADRES hacia las EPS de los recursos asociados a la prestación de servicios no financiados con la UPC.

   Adicionalmente, el Macroproceso permite la transformación de información asociada a los recobros de las EPS y asociada al proceso de conciliación de presupuestos máximos.

   El Macroproceso obtiene información para el proceso de financiación del sistema a partir de diversas fuentes. Estas fuentes proporcionan los recursos necesarios para

garantizar la sostenibilidad financiera y el adecuado funcionamiento del sistema de salud en el país.

Es precisamente el procedimiento asociado a las fuentes de financiación y su interacción con el Macroproceso las que generan el conocimiento inicial para el mismo. Por esta razón, el resumen que se presenta a continuación sobre estas fuentes se constituye en el conocimiento inicial que lo alimenta, el cual se asocia al detalle de información que cada una de estas fuentes aporta para su funcionamiento.

Una de las fuentes de financiación más importantes del SGSSS es el aporte de los afiliados al sistema. A través de las cotizaciones realizadas por los trabajadores y empleadores, se recaudan los recursos que se destinan a financiar la atención en salud de los afiliados. Estos aportes se realizan de acuerdo con las normas establecidas por el Ministerio de Salud y Protección Social.

Otra fuente de financiación son los recursos provenientes del Presupuesto General de la Nación. El Gobierno destina una parte de los ingresos fiscales para fortalecer el SGSSS y garantizar la atención en salud de la población más vulnerable, a través de programas como el Régimen Subsidiado. Estos recursos se asignan de acuerdo con criterios establecidos por el Ministerio de Hacienda y Crédito Público.

Además, existen otras fuentes de financiación, como los recursos provenientes de la participación de las entidades territoriales y los ingresos por concepto de copagos y cuotas moderadoras. Estos recursos se utilizan para complementar el financiamiento del sistema y garantizar la prestación de servicios de salud a los afiliados.

En resumen, las principales fuentes de financiación del SGSSS en Colombia son los aportes de los afiliados, los recursos del Presupuesto General de la Nación, la participación de las entidades territoriales y los ingresos por copagos y cuotas moderadoras. Estas fuentes permiten obtener los recursos necesarios para financiar el sistema de salud y garantizar la atención en salud de la población colombiana y demás fuentes definidas por la ley.

3. Síntesis de la descripción procedimental.

El Macroproceso de financiación inicia con la definición hecha para cada usuario en relación con su pertenencia a alguno de los regímenes tal y como se describió en el Macroproceso anterior, donde también se definen las características y condiciones con las que el sistema respalda la financiación para cada uno de ellos.

Es así que la responsabilidad del proceso de cotización que alimenta esta financiación es vigilada por la Unidad de Gestión Pensional y Parafiscales (UGPP), que es una entidad de la orden nacional adscrita al Ministerio de Hacienda y Crédito Público que adelanta tareas de seguimiento, colaboración y determinación de la adecuada, completa y oportuna liquidación y pago de las contribuciones parafiscales de la Protección Social.

Para los usuarios del régimen contributivo esta obligación tiene que ver con el pago de los aportes y para los usuarios del régimen subsidiado, esta obligación tiene que ver con el reporte de los posibles cambios y novedades que puedan impactar su situación ante el sistema y por tanto que se tramiten de manera adecuada los recursos fiscales transferidos a la ADRES.

En este procedimiento existe solidaridad de los cotizantes, donde los aportes son proporcionales e individuales por cada fuente de ingresos que posean y que cumplan con las condiciones para esta cotización.

Adicionalmente a esta explicación es importante señalar que existen dos mecanismos adicionales para que esta financiación garantice el acceso de los usuarios a los

servicios:

- Recobros por servicios NO PBS y los cuales no se financian con la UPC, y
- Techos o presupuestos máximos.

El primero cuenta con un aplicativo denominado MIPRES donde se consultan los servicios autorizados los cuales, una vez son autorizados y facturados por el médico tratante y pagados por la EPS, generan un recobro de su valor ante la ADRES, generándose un proceso inverso en cuanto a la financiación en comparación con los servicios pagados con la UPC.

El MIPRES es una herramienta tecnológica que implementa el Ministerio de Salud y Protección Social para garantizar el acceso, reporte de prescripción, suministro verificación, control, pago y análisis de la información de las tecnologías en salud no financiadas con recursos de la UPC y servicios complementarios.

En el segundo caso, anualmente el Minsalud define un tope de recursos para financiar servicios que no se encuentran contemplados ni en el PBS ni en el MIPRES, pero necesarios en algunos casos particulares, los cuales pueden ser utilizados por las EPS cumpliendo unos requisitos y unas condiciones especiales para su ejecución, conocidos inicialmente como techos presupuestales y actualmente como presupuestos máximos.

El giro de estos recursos se realiza de manera anticipada a cada EPS, siendo sujeto a posibles ajustes posteriores de acuerdo a la información reportada al Minsalud y a la ADRES.

Para complementar esta descripción, es importante describir el proceso de Compensación como componente conexo al Macroproceso de Financiación.

- Compensación.

Operativamente la Compensación es el proceso mediante el cual el pago total de las cotizaciones obligatorias sea recaudado en cuentas unificadas del sistema o por las EPS, financia las UPC de los afiliados que estén al día en sus pagos en cada una de las EPS.

En esta etapa la ADRES recibe los recursos correspondientes a las cotizaciones de los usuarios del régimen contributivo y los recursos provenientes del sistema de presupuesto general de la nación y del sistema general de participaciones.

La Unidad de pago por capitación (UPC) es el valor anual que se reconoce a las EPS por cada uno de los afiliados al sistema general de seguridad social en salud (SGSSS) para cubrir las prestaciones del Plan Obligatorio de Salud (POS) o Plan Básico de Salud (PBS), en los regímenes contributivo y subsidiado.

La finalidad del giro de las UPC a las EPS es permitir que estas garanticen el acceso a los servicios de salud a sus afiliados y el pago correspondiente a los prestadores de servicios.

El valor de estas unidades UPC es definido anualmente por el Minsalud y es particular para cada usuario, atendiendo a variables tales como la edad, género y lugar de residencia entre otros y se calcula que sea suficiente para cubrir la totalidad de servicios que anualmente es definida por el Minsalud como incluidas en el PBS.

Al tratarse de un valor diferencial por usuario, cada aseguradora recibe por medio de un giro económico adelantado por la ADRES, un monto total asociado a la cantidad y a las características de sus afiliados.

El SGSSS contempla un mecanismo complementario definido para que os prestadores de servicios de salud reciban los recursos que garanticen esta prestación a los afiliados de las EPS, denominado giro directo.

En este proceso, las EPS autorizan el giro directo a los prestadores por los servicios otorgados a sus afiliados. Posteriormente, este valor se descuenta en el proceso de Compensación del valor de UPC que debe girar la ADRES a las EPS de manera periódica.

4. Errores de duplicidad de información.

   En el Macroproceso de Financiación existe información duplicada que se puede generar en el registro de los datos de los afiliados, por lo cual es pertinente mantener un sistema de información robusto.

   La situación generada por el fenómeno denominado "multiafiliación" y que fue descrita anteriormente, genera desbalances tanto en la atención como en las finanzas del sistema. Estos desbalances tienen que ver con posibles errores de doble pago por el registro simultáneo en dos EPS y por esta razón los sistemas de control actúan negando el pago de las UPC a las EPS involucradas hasta tanto no se aclara la situación.

   Lo anterior origina que el usuario multiafiliado no se considere como activo en ninguna de estas EPS involucradas y por esa razón no cuenta con una red para recibir atención.

   Es así que, una persona que se encuentra afiliada a alguno de los regímenes ya sea el contributivo o el subsidiado y de manera simultánea también lo está en algún régimen especial o de excepción, ocasiona la posibilidad de errores en la consistencia y duplicidad de la información, falencias en los procedimientos que se activan automáticamente para los afiliados, afectaciones a la financiación de uno u otro régimen, activación errónea u omisión de los procesos de IVC al ocasionarse un conflicto en la definición de los procesos y entes de control que deben adelantarlos.

   Es importante resaltar que las consecuencias de las prácticas equivocadas de multiafiliación que se presentan en el sistema y que han sido evidenciadas por el equipo de trabajo de esta consultoría, favorecen inconsistencias en otros ámbitos del Macroproceso de financiación.

5. Errores de información oculta, equivocada o inexistente.

   De manera similar al análisis hecho para la información duplicada en los párrafos anteriores, en este Macroproceso existe riesgo de información equivocada, oculta o inexistente que se puede generar por situaciones similares a las anteriormente explicadas.

   La posibilidad de que en las cotizaciones independientes existan múltiples contratos, pero sólo se formalice una declaración y con ella se soporten los aportes en cada uno de los contratos, configura prácticas de elusión.

   La elusión hace referencia al momento en que se realizan los pagos a la seguridad social por un ingreso base de cotización inferior al que realmente corresponde. Esto ocasiona que en las bases de datos se les asigne una categoría más baja a la que debería pertenecer en atención a sus ingresos generando riesgos de financiación para el sistema, en una práctica que deriva en subregistro en las cotizaciones.

   En ese mismo sentido, también se evidencian prácticas de evasión cuando un usuario formaliza su afiliación al régimen subsidiado cuando sus características y su condición definen que debería afiliarse al régimen contributivo.

6. Existencia de un consumo innecesario o excesivo de tiempo y/o recursos, ocasionado por los errores identificados.

Aunque los casos de información duplicada, errónea, oculta o inexistente han ido disminuyendo en la medida en que se aumentan los controles y los cruces de información, el sistema aún presenta falencias en este sentido y por tanto aún se presentan este tipo de desviaciones.

Esta falta de unicidad total en la información almacenada en diferentes bases de datos según se acaba de explicar, afecta procesos asociados a la financiación y genera reprocesos para su conciliación y corrección, como es el caso de las auditorías de la ADRES. También se genera uso excesivo de recursos para lograr identificar las duplicidades e inconsistencias, identificar los recursos que se desembolsaron erróneamente, para generar reportes de inconsistencias de este tipo a cada EPS involucrada, reprocesos para que las EPS puedan validar la situación y activar los mecanismos para que estas reintegren los recursos a la Administradora, para luego obtener efectivamente este reintegro ya sea por traslado de recursos o por cruce con los documentos de Liquidación Mensual de Afiliados (LMA) o valor mensual reconocido a cada EPS desde el régimen subsidiado.

el proceso de compensación se afectaría, teniendo que realizar en algunos casos correcciones al mismo comprometiendo innecesariamente recursos y tiempo para su solución.

7. Contribución de la solución de estos errores al correcto funcionamiento del modelo de gestión del conocimiento.

   La correcta gestión de la información proveniente de las fuentes además del conocimiento que se crea en este Macroproceso, evita la necesidad de reprocesos asociados tanto a la corrección de los errores de conocimiento detallados anteriormente como a la necesidad de corregirlos.

   Algunos de los errores que tradicionalmente se han generado por esta gestión equivocada de cierto tipo de información, ha estado asociada a procesos de multiafiliación, los cuales conllevan a inconvenientes en el sistema tal y como se ha detallado a lo largo de este apartado.

   De manera complementaria, las posibles demoras en la solución de estas inconsistencias también se constituyen en un inconveniente para la consecución adecuada del objetivo, debido a que la dinámica de algunas EPS y las tasas de desaparición de las mismas en Colombia debido precisamente a problemas en su estabilidad financiera, pueden impedir que los dineros que fueron asignados de manera errónea se puedan recuperar fácilmente, generando vacíos fiscales y financieros en su funcionamiento.

   El correcto desarrollo de los mecanismos asociados a la financiación y asignación de recursos (aportes, UPC, MIPRES, Presupuestos máximos) desemboca así mismo en un adecuado flujo de recursos de acuerdo con la normatividad establecida, lo que redunda en la consecución del objetivo definido garantizando la protección financiera del Sistema.

   Lo anteriormente descrito permite afirmar que, la normalización del SGC y su consecuente contribución en la reducción de posibles errores asociados a la información, contribuiría en gran medida a la consecución del objetivo para asegurar los recursos necesarios para garantizar el goce efectivo del derecho a la salud.
