# Supplementary material for: Proposal of a Knowledge Management Model for Complex Systems: Case of the Supervision and Control Subsystem of the Colombian Health System
Source: J Mark Access Health Policy. 2024 Aug 21;12(3):224–51. doi: 10.3390/jmahp12030019 (PMC11348183; doi:10.3390/jmahp12030019)
Supplement: Supplementary file 1 [file jmahp-12-00019-s001.zip › S4 Risk Management Macroprocess Description.pdf]

Macroproceso 4. Gestión Del Riesgo En Salud

1. Objetivo.

   El objetivo de este Macroproceso consiste en lograr el acceso oportuno, efectivo, equitativo, eficiente y de manera sostenible a los servicios cubiertos por el plan de beneficios en salud (PBS) del SGSSS. Teniendo en cuenta que el alcance de este objetivo es el aseguramiento en salud, se enfoca este Macroproceso en la gestión del riesgo primario y técnico, que está a cargo de las EPS.

2. Conocimiento inicial que alimenta el Macroproceso.

   Las necesidades de los afiliados y la información que ingresa al Macroproceso como resultado de estas, pueden catalogarse en diferentes grupos:

   - Historia existente para cada usuario relacionada con los eventos de salud y riesgos individuales.
   - Solicitud de los usuarios para obtener la autorización respectiva y ser incluido en las políticas de atención de urgencias o como parte de tratamientos autorizados para la promoción y prevención de la salud.
   - Necesidades particulares de acceso a tratamientos y procedimientos asociados a la condición de alud de cada usuario.
   - Definición de tipo de servicios autorizados a cada usuario, además de la forma, condiciones y tarifas de pago de estos servicios.

   La información que ingresa al Macroproceso permite el acceso de los usuarios a los servicios del PBS, dado que se posibilita la comunicación sobre las particularidades y las necesidades de cada usuario, para de esta manera establecer de quien es la responsabilidad de asumir la gestión del riesgo.

   Esta información de entrada al Macroproceso de Gestión del riesgo se transforma en la medida en que, durante su utilización, esta se categoriza y se organiza en cuadros de salida para cada usuario, donde las IPS reportan la evolución de los Indicadores de gestión del riesgo en salud a las EPS, asegurando la actualización de la información en cada caso.

   Por otro lado, la información de oportunidad y calidad en la atención que se origina en la prestación de los servicios es reportada al Sistema en cada una de las ocasiones en que existe una interacción entre los usuarios y la IPS asignada.

3. Síntesis de la descripción procedimental.

   El Macroproceso de gestión del riesgo en salud en el SGSSS es fundamental para garantizar la seguridad y el bienestar de los ciudadanos colombianos en términos de salud. A continuación, se describen los pasos que se surten a lo largo de la ejecución procedimental de este Macroproceso, resaltando que estos se ejecutan en la medida en que se hacen necesarios para asegurar la consecución del objetivo de este Macroproceso.

   - Identificación y clasificación de riesgos de los afiliados. La identificación, análisis y clasificación del riesgo de cada afiliado es el punto de partida para la gestión del riesgo en salud, que orienta la organización de la atención según los riesgos identificados, la organización de la red integral de prestadores de servicios de salud requerida y la articulación de las acciones individuales competencia de la EPS con las acciones colectivas y poblacionales que realizan las entidades territoriales y otros sectores.

- Evaluación de riesgos. Una vez identificados los riesgos, se procede a evaluar su probabilidad de ocurrencia y su impacto potencial. Esta evaluación permite determinar la magnitud de cada riesgo y establecer prioridades para su manejo.
- Planificación de estrategias de gestión del riesgo. La estratificación de los riesgos en salud identificados para los afiliados permite definir en primera instancia los modelos y estrategias para su atención.

  Con base en la evaluación de riesgos, se desarrollan estrategias y planes para mitigar, prevenir o controlar los riesgos identificados. Estas estrategias pueden incluir medidas de prevención, acciones correctivas, capacitación del personal, adquisición de recursos adicionales, entre otras.
- Implementación de medidas de control. Una vez establecidas las estrategias de gestión del riesgo, se lleva a cabo su implementación. Esto implica la ejecución de las medidas de control planificadas para minimizar los riesgos y garantizar la seguridad y calidad de los servicios de salud.
- Monitoreo y seguimiento. Es importante llevar a cabo un monitoreo continuo de las medidas de control implementadas para evaluar su efectividad y realizar ajustes si es necesario. También se realiza un seguimiento de los nuevos riesgos que puedan surgir y se toman las medidas correspondientes para abordarlos adecuadamente.
- Comunicación y participación. La gestión del riesgo en el SGSSS también implica una comunicación clara y efectiva con todos los actores involucrados, incluyendo el personal de salud, los pacientes y las autoridades competentes. Se fomenta la participación de todas las partes interesadas en la identificación y gestión de riesgos.

  En resumen, el proceso de gestión del riesgo en el SGSSS involucra la identificación, evaluación, planificación, implementación, monitoreo y comunicación de medidas para minimizar los riesgos y garantizar la seguridad y calidad de los servicios de salud. Es un proceso continuo y fundamental para asegurar la atención médica adecuada y proteger la salud de los ciudadanos colombianos y contempla acciones desde la óptica de servicios de salud, financiero y administrativo, los cuales se relacionan a continuación.

  De esta gestión del riesgo se despenden las obligaciones financieras de pago a los prestadores.

a) Salud .

- Estructurar la red de prestación.

  La organización de la red de prestadores de servicios y proveedores de tecnologías en salud en una EPS por territorio, parte de la identificación de los riesgos de los afiliados y la clasificación de los mismos por grupos de riesgos, para en un proceso de acuerdo de voluntades se establecen los servicios a prestar, la forma de pago y los mecanismos de control de la calidad con los que se monitorean estos procedimientos, además de la documentación que deberá presentarse como soporte de cumplimiento a las condiciones del contrato.

  La actualización de la red de servicios es un proceso continuo y se convierte en un flujo de información a los usuarios, donde se les notifican las opciones de prestación de servicios de la que disponen.

  Esa estructuración resulta en redes de primer nivel y de niveles complementarios, incluyendo hospitalizaciones y apoyos terapéuticos, diagnósticos y la entrega de medicamentos e insumos ambulatorios requeridos por los pacientes.

- Garantizar el acceso, referencia y contra referencia.
  Los a acuerdos de voluntades pactados con la RISS define los estándares los estándares de solicitud de servicio, los mecanismos de acceso adecuados para esta prestación y tanto el alcance como las limitaciones que cada prestador ofrece, donde estas últimas deben ser suplidas con un esquema de referencia que indique las alternativas de prestadores disponibles.
- Suministro de tecnologías y medicamentos.
  Se establece un acuerdo de voluntades con proveedores de tecnologías médicas, de medicamentos donde se establece el detalle de estos servicios, las cantidades, precios y precios, donde se define que esta red de prestadores hace parte disponibles para los usuarios.
  Por otro lado, la EPS define qué tipos de servicios y medicamentos no son objeto de autorización previa según su modelo de atención, por lo que una vez solicitados por los profesionales a cierto tipo de usuarios donde solo hace falta la identificación de estos y su pertenencia al sistema para el suministro de estos.
  En los casos en que se necesita la autorización, la aseguradora define los procedimientos necesarios para adelantarla y de esta manera recibir la tecnología o medicamento asignado por el profesional.
  Posteriormente, los prestadores han definido con las aseguradoras la remisión de información asociada a los montos económicos correspondientes a lo anterior, además de los soportes relacionados con la entrega o prestación efectiva de estos insumos o servicios.
  El acceso a los servicios y el suministro de tecnologías y medicamentos originan los Registros Individuales de Prestación de Servicios (RIPS) remitidos a las EPS y al Minsalud como soporte de estas prestaciones.
- Auditorías de calidad y concurrencia.
  De acuerdo con el tipo de servicio prestado existen diferentes esquemas de evaluación antes, durante y posterior a la ejecución.
  Las auditorías concurrentes involucran una revisión en el momento mismo de la prestación, siendo los casos más comunes los correspondientes a los procesos de hospitalización.
  En estos casos los auditores, concurren durante el proceso para revisar la prestación, la historia médica, la asignación de medicamentos, el dictamen médico, donde en caso de presentarse algún tipo de demora en esta asignación gestionan su solución para aminorar los riesgos al paciente. De esta manera mejoran la oportunidad en la atención y por tanto la calidad de esta.
- Garantía de la calidad de la atención.
  Para gestionar esta garantía, se definieron 4 componentes del Sistema Obligatorio de Garantía de la Calidad.
  El primero es la Habilitación de Servicios que implica que las EPS no pueden contratar servicios que no tengan autorización del Estado. Esto garantiza que todos los prestadores hayan obtenido la habilitación para los servicios que pueden ofrecer.
  El segundo es el Plan de Auditoría para el Mejoramiento de la Calidad (PAMEC), que funciona como un sistema de mejoramiento continuo asociado a los procedimientos de los prestadores y de las aseguradoras.
  En tercer lugar se define un proceso para aseguramiento de información para la calidad, el cual contiene toda la documentación y las normas que definen los indicadores de prestación que las IPS deben remitir a las EPS y a los entes territoriales para posteriormente ser remitidos al Minsalud, que

contiene cifras sobre aspectos tales como la oportunidad de la asignación de citas, oportunidad en la atención, mortalidad general y sobre mortalidad materna entre otros y que conforman el esquema de gestión de la calidad.
En cuarto lugar se encuentra el sistema de estándares superiores contenidos en el Sistema de Acreditación autónomos de la red de prestadores, la cual permite la identificación de niveles óptimos de los servicios en comparación con otros componentes del sistema, situación que se convierte en un diferencial al interior del Sistema.

- Promoción y mantenimiento de la salud.
  El sistema ha definido actividades denominadas de "Demanda inducida", donde los prestadores adelantas procedimientos de búsqueda de población en riesgo (asociados a los antecedentes, a criterios de riesgo y a la edad, entre otros), para brindar servicios de prevención y atención temprana que aminoren los riesgos para la población.
  Este proceso genera unos Registros de prestación de servicios y de procesos de prevención, los cuales son utilizados no solo para el proceso de aseguramiento sino también para la definición de políticas para el mejoramiento en los niveles de salud para la población.

b) Financiero

- Contratar la Red.
  Para garantizar el Plan Obligatorio de Salud a sus afiliados, las Entidades Promotoras de Salud prestarán directamente o contratarán los servicios de salud con las Instituciones Prestadoras y los profesionales. Para racionalizar la demanda por servicios, las Entidades Promotoras de Salud podrán adoptar modalidades de contratación y pago tales como capitación, protocolos o presupuestos globales fijos, de tal manera que incentiven las actividades de promoción y prevención y el control de costos. (artículo 1 Ley 100 de 1993
- Pagar los servicios prestados a cada usuario.
  Las EPS de ambos regímenes, pagarán los servicios a los Prestadores de Servicios de salud habilitados, mes anticipado en un 100 % si los contratos son por capitación. Si fuesen por otra modalidad, como pago por evento, global prospectivo o grupo diagnóstico se hará como mínimo un pago anticipado del 50 % del valor de la factura, dentro de los cinco días posteriores a su presentación. En caso de no presentarse objeción o glosa alguna, el saldo se pagará dentro de los treinta días (30) siguientes a la presentación de la factura, siempre y cuando haya recibido los recursos del ente territorial en el caso del régimen subsidiado. (artículo 13 Ley 1122 de 2013).
- Recaudo de cuotas moderadoras y copagos.
  Al momento en que un usuario utilice un servicio o reciba un medicamento o tecnología y de acuerdo con su régimen y categoría definidos en el Macroproceso de afiliación, adquiere o no la obligación de pagar un copago o una cuota moderadora. Este valor es recaudado por el prestador de salud y se descuenta de los valores de la cuenta médica que se factura a la EPS correspondiente al servicio, medicamento o tecnología.
- Control de gasto – auditoría de cuentas.
  Esta auditoría procura identificar las irregularidades en los procesos de facturación desde la perspectiva de la pertinencia médica, la calidad, el cumplimiento de los acuerdos contractuales definidos y los parámetros generales de la atención en salud, resguardando el control financiero del gasto y el flujo de caja del Asegurador.
- Recobros.

La EPS así como gestiona los recursos asignados a través de la UPC para el pago de los servicios y tecnologías cubiertos en el plan de beneficios, desde el año 2020 también debe realizar esta gestión con los recursos asignados por presupuestos máximos para el pago de los servicios y tecnologías no PBS, los cuales igualmente será girados por la ADRES, aplicando la metodología definida por el Minsalud para el reconocimiento de estos recursos por EPS. Por este motivo, en la actualidad, las EPS no deben realizar recobros a la ADRES excepto en casos de servicios y tecnologías excluidos, servicios sociales complementarios o los que ordenan un tratamiento integral ordenados en fallos de tutela y providencias judiciales (parágrafo del artículo 5° y el numeral 10.8 del artículo 10 del Decreto 521 de 2020 fueron modificados por el Decreto 1810 de 2020)
Mediante el Decreto 521 de 2020 se reglamenta el artículo 237 de la Ley 1955 de 2019 y se establecen los criterios para la estructuración, operación y seguimiento del saneamiento definitivo de los recobros por concepto de servicios y tecnologías de salud no financiados con cargo a la UPC del Régimen Contributivo.
Mediante el Decreto 521 de 2020 se reglamenta el artículo 237 de la Ley 1955 de 2019 y se establecen los criterios para la estructuración, operación y seguimiento del saneamiento definitivo de los recobros por concepto de servicios y tecnologías de salud no financiados con cargo a la UPC del Régimen Contributivo.

c) Administrativo

- Pago de prestaciones económicas.
  En el caso del otorgamiento de incapacidades por enfermedad general, o licencia de maternidad o paternidad emitidas a los usuarios cotizantes del régimen contributivo, la licencia correspondiente genera una serie de implicaciones de tipo financiero.
  Según el Decreto 780 de 2016, los empleadores asumen 100 % las prestaciones económicas correspondientes a los 2 primeros días de incapacidad originada por enfermedad general, y las Entidades Promotoras de Salud a partir del tercer (3) día reconocerán las incapacidades con el 66.67 % para los primeros 90 días y a partir del día 91 hasta 180 en cuantía de 50 % de salario total de liquidación.
  El empleador debe corroborar la información y efectuar el pago del salario al trabajador durante su incapacidad. Adicionalmente, el empleador debe efectuar el trámite de transcripción ante la EPS, para luego realizar el recobro del dinero pagado al trabajador.
  En cualquiera de los casos anteriores la empresa le reporta al sistema esta situación, para que por medio del proceso de recobro le sean reconocidos estos montos pagados al empleado por parte de la EPS. Este reconocimiento, en el caso de la incapacidad por enfermedad general se hace con cargo a una cuenta definida en ese sentido por la ADRES y la cual se financia con un porcentaje específico del total de aportes hechos al sistema y cuyo monto y porcentaje está definido por la ley. En el caso de las licencias de maternidad o paternidad, el empleador notifica esta situación ante la EPS y esta a su vez genera un proceso de cobro a la ADRES por este concepto.

4. Errores de duplicidad de información.

   En el Macroproceso de Gestión del Riesgo existen diferentes situaciones evidenciadas por el equipo de trabajo de esta consultoría, tiene que ver principalmente con la

multiplicidad de información y de bases de datos, que en algunas ocasiones no surgen de un origen común presentándose duplicidades desde el origen, o en otras ocasiones si se originan en una misma fuente pero son distribuidas a diferentes prestadores quienes las modifican en razón a sus propios procedimientos operativos, pero no existe un procedimiento ni la normatividad necesaria para que cada cierto tiempo, se logre una depuración y unificación de esta información, generándose errores y falta de concordancia en datos de algunos usuarios, a pesar de tratarse de la misma persona.

La ausencia de un sistema único de información, hecho que ha sido advertido en diferentes ocasiones por directivas del sector oficial, conlleva a que exista tanta información diferente asociada a un mismo usuario, en repositorios independientes que son modificados por entidades distintas.

La ausencia de normalización de los registros en los casos en que los usuarios modifican su EPS también genera duplicidades ante la posibilidad de que exista información en diferentes bases por cada asegurador y en la IPS primaria de cada una de las EPS en las que ha estado afiliado.

5. Errores de información oculta, equivocada o inexistente.

Una de las situaciones más comunes de información equivocada y oculta evidenciadas en el análisis de esta consultoría, tiene que ver con los procedimientos disponibles para los usuarios para contactar a las EPS y solicitar atención en salud en los diferentes tipos que se relacionan con la cobertura contratada y que en la mayoría de los casos está asociada con las obligaciones de cumplir estándares de oportunidad por parte de los aseguradores y por parte de los prestadores.

Esta situación tiene que ver con la ausencia de una trazabilidad real de estos procedimientos desde el momento en que el usuario se pone en contacto por primera vez con alguna de las entidades enunciadas, para lograr acceder al sistema. Dado que existen estándares mínimos de oportunidad, en varios casos se evidencia que la información y los tiempos de atención y autorización que se reportan a los organismos de control, no son reportados desde la primera ocasión en que el usuario solicita este contacto sino desde el momento en que el servicio se autoriza.

Esta información y los resultados de los estándares de acceso asociados, no se ajustan a la realidad y falsean el propósito de los indicadores de atención. El hecho de no iniciar el análisis y la medición desde que se registra la primera intención de solicitud de servicios contratados por el usuario hasta el momento en que la obtiene, sino de iniciar esta medición desde el momento en que el acceso es autorizado, no muestra la realidad de los tiempos y de la oportunidad utilizados por los aseguradores y/o prestadores, sino que mostraría en todos los casos resultados ajustados a la normatividad, sin importar de que se trate de información equivocada y oculta.

De manera complementaria al análisis anterior, también se accedió al análisis de las PQRS y a la posibilidad de que dependiendo la manera en cómo se resuelven y originan respuestas, también pueden generar información que no se ajusta a la realidad y esto conlleva a la existencia de información errónea, oculta o sencillamente inexistente. Cuando las respuestas a estas PQRS no resuelven de fondo estas solicitudes, sino que se envían utilizando respuestas pre formateadas con mensajes genéricos para cumplir unos estándares de cumplimiento, existe información errónea que oculta los tiempos reales que se utilizan para resolver cada uno de los casos.

Otra información inexistente en algunas EPS está relacionada con la ausencia de construcción completa y correcta de la caracterización de la población afiliada, que más allá de la información sociodemográfica y general, carece de información sobre

factores de riesgo, grupo de riesgo, diagnóstico y otros aspectos relacionados, necesaria para que permita a la EPS conocer realmente su población para la adecuada gestión de los riesgos de ésta.

Igualmente, la poca adherencia a las guías de Práctica clínica en los prestadores genera en algunos casos errores en los diagnósticos que al ser reportados a la EPS no permite realizar la identificación correcta del riesgo de los afiliados y su consecuente clasificación en grupos de riesgos, cuando aplica, demorando o incluyendo erradamente a un usuario en un grupo de riesgo que no pertenece. Esta misma, situación puede generar errores en el plan de manejo de los pacientes, implicando su atención mayores costos y riesgos en salud de los necesarios.

Por parte de las EPS se evidencia ausencia de información clara y real sobre la prestación efectiva de los servicios y tecnologías en salud a sus afiliados, fundamentales en lo débiles procesos de auditoria o seguimiento a la red de prestadores contratada.

En el ámbito de conformación de la red de prestadores, en algunos casos la información no actualizada en e REPS de los servicios y la capacidad real de los prestadores, puede desencadenar en la no conformación de la red conforme a las necesidades y demanda de los afiliados. Adicional a esto, en los acuerdos de voluntades no se deja explícito desde su suscripción toda la información que es requerida para el adecuada ejecución del mismo.

Por otro lado, la ausencia de unificación procedimental en el Sistema y la diferencia en que la reglamentación contable es aplicada en el caso de los copagos y de las cuotas moderadoras, originándose una falta de concordancia en diferentes actores del sistema, dado que los mismos son considerados en algunas ocasiones como anticipos o pago anticipado en el pago de servicios y en la facturación asociada y en otras ocasiones, estos son considerados como un menor valor en las cuentas periódicas. Aunque estas dos modalidades pueden ser autorizadas contablemente, la ausencia de unificación en el tratamiento que se les da ocasiona que los resultados no puedan ser unificados ni considerados en la misma forma para todos los actores involucrados, situación que podría considerarse como información no equivalente y equivocada, atendiendo a la óptica que se elija para su análisis.

Una situación similar se detectó en el caso de algunas autorizaciones médicas, donde por motivos que no son justificados por los aseguradores o por los prestadores, son emitidas a los usuarios para su ejecución. Sin embargo, cuando se inicia su trámite, posibles errores, falencias o inconsistencias son detectadas en el procedimiento ejecutado, lo que hace necesario que el usuario reinicie alguna de las etapas que ya surtió para lograr la autorización acertada. Esta situación que ocurre en diversos casos genera también información errónea u oculta a pesar de que en los indicadores de oportunidad y de respuesta no se reflejen.

Complementando lo anterior, también se registra el caso de las entregas parciales y/o en diferentes fechas de medicamentos que son recetados por el personal médico. En diferentes ocasiones se reporta la oportunidad en la entrega de estos, sin importar si la orden se despachó en su totalidad, en diferentes fechas con entregas parciales o si definitivamente queda alguno de los medicamentos sin entregar. Dado que el reporte no contiene el detalle de estas posibilidades, también se considera información errónea e incompleta. En el caso de las entregas parciales existen casos en los cuales el reporte del pendiente que se originó con la primera entrega parcial, no se registra en la fecha de la entrega incompleta sino en la fecha de la entrega definitiva. Esto supone que el pendiente de entrega se resolvió el mismo día en el que se originó, lo cual no responde a la realidad.

6. Existencia de un consumo innecesario o excesivo de tiempo y/o recursos, ocasionado por los errores identificados.

   La información duplicada, equivocada, oculta o inexistente supone en sí misma la utilización de recursos y de tiempo para solucionar las falencias en el sistema que se ocasionan por su aparición, los cuales no se utilizarían si la información no presentara este tipo de desajustes.

   Dado que como se explicó anteriormente esta situación genera reprocesos que generalmente deben ser asumidos por el usuario y teniendo en cuenta que en cierta cantidad de casos la simple manifestación por parte de los mismos no es suficiente para lograr una corrección al respecto, es común que se necesite acudir a canales diferentes a los establecidos e incluso a la vía jurídica, lo cual redunda es estos consumos innecesarios.

   Otra situación en la cual ocurren estaos inconvenientes, tiene que ver con el no cumplimiento de las condiciones de atención integral, particularmente en los casos en los que las IPS no pertenecen a la misma agrupación de la EPS elegida. En estos casos y auqnue debería ser la EPS la que se asegure de que la atención, los procedimientos y la entrega de medicamentos se cumple, a menudo es el mismo usuario el que debe adelantar diferentes procesos en cada una de las entidades para lograrlo.

   La utilización de herramientas y las acciones tales como los derechos de petición, las tutelas, la asistencia a juzgados, los recursos jurídicos, la radicación de PQRS

   El no cumplimiento en los procedimientos asociadas a las soluciones efectivas de desviaciones relacionadas en el análisis anterior, siempre resultará en la necesidad de adelantar actuaciones adicionales a las establecidas normativamente y al consecuente uso innecesario o excesivo de tiempo y de recursos en los mismos. Es importante aclarar que ese uso innecesario y ocurre para las partes involucradas, desde el usuario que debe interponerlas hasta las oficinas encargadas en las aseguradoras y en las prestadoras que deben solucionarlas, además de las instancias jurídicas que se incluyen en el proceso.

   Es importante destacar que en algunas ocasiones la gestión misma del riesgo se ve afectada por este uso innecesario y excesivo de tiempo y recursos, dado que los diagnósticos casi nunca son estáticos en razón a la misma evolución de los diagnósticos y en algunos casos, incluso atentan contra las condiciones de salud de los pacientes a causa de la atención a destiempo para su solicitud o incluso la ausencia de soluciones, lo que normalmente afecta sus propias condiciones de salud.

7. Contribución de la solución de estos errores al correcto funcionamiento del modelo de gestión del conocimiento.

   La gestión efectiva del sistema de gestión del conocimiento asociado a la gestión del riesgo en salud es una condición necesaria para asegurar atención oportuna y para garantizar la calidad en salud de los usuarios.

   Aspectos como evitar reprocesos innecesarios, retrasos en la respuesta, asegurar la atención oportuna, la disminución de impedimentos para obtener la atención en salud que el SGSSS ha definido para la población, beneficia a la sociedad. Estas condiciones podrían mejorar los resultados para los cuales se define la gestión del riesgo.

   Dado que esta gestión del riesgo tiene diferentes actores para garantizar el éxito de la misma, la gestión adecuada del conocimiento permitiría la ausencia de situaciones atípicas en algún actor del sistema que pueda afectar a otros eslabones en la cadena.

   También permitiría la toma oportuna de decisiones en términos de salud y la definición de políticas públicas mucho más efectivas que las existentes. Los entes de control

y el regulador también tendrían la posibilidad de corregir posibles desviaciones que afecten lo anteriormente descrito, y podría activar controles de manera temprana para su solución.

En los casos en los cuales la información correcta permita conocer si los problemas de salud de los usuarios pueden tener diagnósticos unificados y como consecuencia se hace posible autorizar tratamientos acertados y la toma de decisiones oportunas en esta materia.
