# Supplementary material for: Proposal of a Knowledge Management Model for Complex Systems: Case of the Supervision and Control Subsystem of the Colombian Health System
Source: J Mark Access Health Policy. 2024 Aug 21;12(3):224–51. doi: 10.3390/jmahp12030019 (PMC11348183; doi:10.3390/jmahp12030019)
Supplement: Supplementary file 1 [file jmahp-12-00019-s001.zip › S5 Surveillance, Inspection and Control Macroprocess Description.pdf]

Macroproceso 5. Vigilancia, Inspección y Control

1. Objetivo.

   El objetivo del Macroproceso de Vigilancia , Inspección y Control puede analizarse respecto a las tres dimensiones que la componen: en su función administrativa de inspección su objetivo es el de solicitar información de las personas objeto de supervisión, así como de practicar visitas a sus instalaciones y realizar auditorías y seguimiento de su actividad; en su función de vigilancia se refiere a las acciones de advertencia, prevención y orientación encaminadas a que los actos del ente vigilado se ajusten a la normatividad que lo rige: finalmente en su función de control permite ordenar correctivos sobre las actividades irregulares y las situaciones críticas de orden jurídico, contable, económico o administrativo.

   El artículo 35 del capítulo VII de la Ley 1122 de 1995, define las funciones de Vigilancia, Inspección y Control de la siguiente forma:

   La vigilancia consiste en la atribución de la Supersalud para advertir, prevenir, orientar, asistir y propender porque las entidades encargadas del financiamiento, aseguramiento, prestación del servicio de salud, atención al usuario, participación social y demás sujetos de vigilancia de la Superintendencia Nacional de Salud, cumplan con las normas que regulan el Sistema General de Seguridad Social en Salud para el desarrollo de éste.

   - La inspección es el conjunto de actividades y acciones encaminadas al seguimiento, monitoreo y evaluación del Sistema General de Seguridad Social en Salud y que sirven para solicitar, confirmar y analizar de manera puntual la información que se requiera sobre la situación de los servicios de salud y sus recursos, sobre la situación jurídica, financiera, técnica-científica, administrativa y económica de las entidades sometidas a vigilancia de la Superintendencia Nacional de Salud dentro del ámbito de su competencia. Son funciones de inspección entre otras las visitas, la revisión de documentos, el seguimiento de peticiones de interés general o particular y la práctica de investigaciones administrativas.
   - El control consiste en la atribución de la Supersalud para ordenar los correctivos tendientes a la superación de la situación crítica o irregular (jurídica, financiera, económica, técnica, científico-administrativa) de cualquiera de sus vigilados y sancionar las actuaciones que se aparten del ordenamiento legal bien sea por acción o por omisión.

   Como marco normativo dentro del cual se desarrolla este Macroproceso hace referencia a la Ley 222 de 1995 de la Superintendencia de Sociedades (Supersociedades), a la Ley 1122 de 2007 (Supersalud) y a la Ley 1493 de 2011 (Dirección Nacional de Derechos de Autor), la Ley 1949 de 2019 (Minsalud), el artículo 2 de la Ley 1966 de 2019 (Minsalud) y el Decreto 1080 de 2021 (Supersalud).

2. Conocimiento inicial que alimenta el Macroproceso.

   Este Macroproceso contempla conocimiento asociado a las condiciones de habilitación de las entidades responsables de la operación del aseguramiento en salud.

   Basándose en los requisitos o condiciones normativas contempladas en la legislación referida, la Supersalud realiza la verificación para determinar el ingreso y permanencia de una EPS en el SGSSS.

   Desde el proceso de Regulación, el Minsalud ha definido las condiciones que las EPS deben cumplir para su autorización, habilitación y permanencia en el Sistema.

Para iniciar el proceso, la entidad interesada en conformarse como EPS presenta una solicitud que soporte el cumplimiento de los requisitos exigidos en el marco legal con el propósito de obtener la autorización de funcionamiento.

La Supersalud verifica el cumplimiento de los requisitos de ingreso y si estos se cumplen, se genera la resolución de autorización de funcionamiento y se le asigna un código a la EPS.

Las EPS presentan los estados financieros al final de cada ejercicio contable a la Junta Directiva y si estos son aprobados, son enviados a la Supersalud para análisis y revisión. En caso de no encontrarse ninguna inconsistencia, se autoriza la puesta en consideración de la Asamblea General de socios, accionistas o asociados, como máximo órgano decisorio. Una vez estos estados sean aprobados por este último órgano, se permite la publicación en los canales definidos como de amplia circulación y, además se envía copia del acta de dicha asamblea a la Supersalud para su verificación y confirmación de que los estados financieros aprobados por la Asamblea corresponden a los que anteriormente habían sido autorizados por la Supersalud para presentación en dicha asamblea.

Las EPS deben presentar reportes periódicos trimestrales (financieros, administrativo, técnicos y científicos) a la Supersalud. Esta información es analizada y de acuerdo con los estándares establecidos por el Minsalud, se cataloga el nivel de riesgo para cada una de estas dimensiones. Los reportes periódicos trimestrales enunciados anteriormente, incluyen tutelas, PQRS e indicadores de calidad en la atención en salud.

En caso de que los indicadores de permanencia de orden financiero sean catalogados como de no cumplimiento, la EPS sería sujeto de la aplicación de medidas especiales atendiendo al nivel de riesgo identificado y como resultado se activa el Sistema de Gestión y Control de Medidas Especiales – FÉNIX, que incluye información sobre el derecho a la salud (tutelas), indicadores de gestión del riesgo en salud, indicadores de gestión de la atención en salud e indicadores de materialización del riesgo en salud, además de los financieros.

De manera no regular, la Supersalud puede tener conocimiento de actuaciones no apropiadas en materia de gestión administrativas por parte de las EPS, por comunicaciones originadas por terceros prestadores de servicios de salud, usuarios, la Contraloría General de la República (CGR) u otras entidades oficiales de Vigilancia, Inspección y Control (IVIC).

3. Síntesis de la descripción procedimental.

Se ha definido que, en el marco de la solicitud de autorización de funcionamiento, una persona jurídica que manifiesta su intención de constituirse como EPS, radica la documentación requerida por la normatividad vigente y que a su juicio soporta el cumplimiento de las condiciones definidas en las normas en términos de capacidad técnico-administrativa, capacidad tecnológica y científica, gobierno organizacional y condiciones financieras para la autorización de habilitación y funcionamiento.

Una vez la Supersalud verifica el cumplimiento, le da la autorización a la persona jurídica, para funcionar como una EPS y le asigna un código que la identificará en el Sistema.

Una vez entra en funcionamiento, la EPS debe iniciar el proceso para cumplir los requisitos de permanencia.

La verificación de los requisitos de permanencia, que incluye el análisis periódico y permanente por parte de la Supersalud de los reportes de información sobre la

capacidad técnico-administrativa, la capacidad tecnológica y científica, el gobierno organizacional y las condiciones financieras y de solvencia de las EPS.

En caso de que los resultados de los indicadores financieros y de solvencia presentados por las EPS no se ajusten a los estándares definidos para su permanencia, la Supersalud solicitará un plan de mejoramiento. Si esta situación es reiterada, se analizará la necesidad de una medida de vigilancia especial.

En caso de presentarse además un incumplimiento en alguno de los otros requisitos, la Supersalud tiene la potestad de generar una alerta de incumplimiento que derive en un requerimiento a la EPS para que elabore y presente un plan de mejora que pueda subsanar las falencias evidenciadas.

La Supersalud emite resoluciones y circulares donde le da lineamientos a los vigilados sobre los reportes que debe realizar, así como acciones para dar cumplimiento por parte de los actores.

La inspección la realiza la Supersalud a las EPS a través de la revisión y análisis de la información reportada por cada entidad, la vigilancia la realiza cuando evidencia falencia y por lo tanto debe realizar mayor seguimiento, solicitar información complementaria, y cuando el riesgo por incumplimiento de indicadores financieros (principalmente) genera estrategias de control como son las medidas especiales, procesos administrativos y sanciones.

4. Errores de duplicidad de información.

   En este Macroproceso existe información duplicada que se puede generar por dos situaciones diferentes. En primer lugar, el sistema se alimenta por información que se puede haberse duplicado desde la fuente, perteneciente a otros Macroprocesos del SGSSS. En otras ocasiones, es el mismo funcionamiento de este Macroproceso el que las origina. A continuación, se explicarán las dos categorías.

   En la primera categoría y como contexto, se puede anotar que el sistema adolece de una fuente unificada de canalización para el ingreso o generación de información al mismo sistema, situación que se evidenció el equipo de trabajo de esta consultoría en diferentes casos, algunos de los cuales se reportan a continuación.

   - Una situación en donde dos actores del sistema generan la misma información acudiendo a fuentes diferentes o a la misma en diferentes momento, cuando algún contenido de esta información pudo haberse transformado, complementado o modificado, situación que ocurre cuando la ADRES realiza una publicación de afiliados por EPS con periodicidad mensual de manera simultánea a la que existe en el SISPRO como fuente oficial , una vez ya se ha adelantado el proceso de compensación y sus resultados han sido validados con la base de datos de la Registraduría Nacional. Esta situación puede ocasionar posibles duplicidades y confusiones tanto en el proceso de IBC, como en los casos en que se deben toma decisiones con base en este insumo.
   - Otro posible dato de inconsistencia por duplicidad se presenta en el reporte trimestral de los indicadores de calidad de las IPS que aplican a cada una por los servicios prestados de acuerdo con la Resolución 256 del 2016 (efectividad, gestión del riesgo, experiencia de la gestión y seguridad) y que se deben remitir al SISPRO del Minsalud. Existe también para las IPS la obligación de reporte a las EPS para que las mismas consoliden la correspondiente a todas las prestadoras que tiene contratadas y reporten al mismo sistema. El equipo de trabajo de esta consultoría ha evidenciado que la información consolidada en niveles de agrupación de EPS

o Departamento no siempre es equivalente a la consolidación de la información que las IPS reportaron de manera individual.

- De la misma forma se evidenció que existe duplicidad en la información en algunos casos en que los usuarios o beneficiarios del sistema son registrados en el mismo bajo una categoría y luego, cuando su situación cambia y son registrados en una nueva, pero no se elimina el registro anterior. Un ejemplo es el de los nacidos vivos, los cuales son registrados inicialmente en el RUAF asociados al documento de identidad del padre o de la madre y con un dígito adicional que los identifica, y donde este registro permanece en la base de datos a pesar de que con el paso del tiempo la misma persona sea registrada con un nuevo documento de identidad asignado de acuerdo con la legislación (NUIP del Registro Civil de Nacimiento, Tarjeta de Identidad o Cédula de Ciudadanía). Se han evidenciado casos, sobre todo en la ruralidad, donde las bases de datos no son depuradas y se presenta este riesgo.

En la segunda categoría la duplicidad de información originada en este mismo Macroproceso puede presentarse cuando en su procedimiento utiliza diferentes fuentes de información que no han sido depuradas o unificadas anteriormente y forman parte de diferentes etapas del proceso. El equipo de trabajo de esta consultoría evidenció algunos de estos casos los cuales se reportan a continuación.

- En diferentes casos donde alguno de los actores con acceso a las bases de datos ejecuta procesos de ingreso, consulta o modificación de cierta información en alguno(s) de los módulos de los diferentes sistemas utilizados en el SGSSS, al terminar su proceso y luego de su utilización o modificación, graba esos cambios en el sistema como información válida y única, sin tener procesos de depuración.
- Las fuentes oficiales para indicadores de gestión en salud (SIVIGILA, SISPRO - indicadores de calidad) no son datos depurados ni validados, lo cual pueden afectar los resultados para una EPS.

En la mayoría de estos casos no existen procedimientos que validen si la misma ya fue consultada para alguno de los actores y se encontraba almacenada. Al no hacerse este proceso de depuración, es posible que se registre nuevamente información que ya había sido registrada anteriormente, adicionándole nuevos datos. Dado que la fragmentación de sistemas no permite consultar en tiempo real si ya existe información para alguno de los actores (usuarios, procedimientos, solicitudes, IPS, etc.), este nuevo ingreso genera nuevos registros cuando en realidad debió haberse modificado alguno ya existente, generándose en estos casos errores de duplicidad e inconsistencia de manera simultánea.

- Otro ejemplo típico de duplicidad e inconsistencia de la información se presenta comúnmente en los procesos financieros, de cobro y pago de facturas y cuentas generadas en la interacción de diferentes actores, sobre todo entre las IPS y las EPS. El equipo de trabajo de esta consultoría evidenció un sinnúmero de casos en los cuales la información avalada por cada una de estas instancias presenta diferencias, que en algunos casos es significativa. Esta situación se generó en diferentes casos porque existían diferentes documentos o facturas para la misma situación, situación que en algunos casos activa procesos de conciliación.
- En otros casos y en razón a la variedad de figuras societarias existentes en la legislación colombiana, también existen diferencia en los entes de control que aplican para unas y otras entidades. Es así que en materia tributaria existe

competencia de la DIAN, pero en cuanto a otros aspectos financieros y contables existen otros entes de control atendiendo a esta variedad. Algunos ejemplos corresponden a las entidades del grupo solidario donde existe competencia de la Superintendencia de la Economía Solidaria (Supersolidaria) o para las entidades relacionadas con las Cajas de compensación familiar existe competencia del Subsidio Familiar (Supersubsidio), entre otras. Esta diferenciación de los entes de control para las diferentes tipologías de empresas hace necesario que además de activar el procedimiento definido al inicio de este Macroproceso, también deban generarse reportes de diferente índole para estos entes de control, los cuales deben remitirse en diferentes formatos y haciendo uso de diferentes medios. Esta multiplicidad de información, genera un alto riesgo de duplicidad o equivocación en la misma información.

- Continuando con este análisis en materia financiera y contable, equipo de trabajo de esta consultoría constató que un caso típico de información duplicada está asociada a las situaciones de cartera originada en cualquiera de las etapas del SGSSS, donde la información que reportan las entidades puede ser diferente a la del Minsalud y a su vez, puede ser diferente a la de la Supersalud. Aunque estas diferencias se intentan sanear en diferentes procesos de conciliación, mientras no se logre un resultado adecuado que homogenice las cifras, siempre existirá riesgo de duplicidad.
- Complementando este análisis de la información asociada a la cartera, también se evidenció falta de concordancia en los períodos a los que corresponde la publicación de esta información y/o a la periodicidad en que es emitida por los entes. Se evidenciaron casos y tipologías de reportes en que el correspondiente al Minsalud es trimestral y el equivalente emitido por la Supersalud es mensual. Esta situación permite que exista riesgo de información duplicada o errada, la cual es difícil de detectar por esta diferencia de categorías y posiblemente en los formatos y la estructura de consolidación.

La situación generada por la emisión de normatividad asociada a algunos de las etapas del Macroproceso que desconoce la existencia de normatividad anterior que ha tratado parcial o totalmente el mismo tema y donde no se tiene el suficiente cuidado de advertir que reemplaza las anteriores, ocasiona que exista diferente normatividad sobre los mismos temas, posibilitando diferentes interpretaciones en actores diferentes sobre la misma temática, situación que a su vez genera posibilidad de duplicidad en la información.

5. Errores de información oculta, equivocada o inexistente.

De manera similar al análisis hecho para la información duplicada en secciones anteriores, en el Macroproceso d IVC existe riesgo de información equivocada, oculta o inexistente que se puede generar por las mismas dos situaciones explicadas. En primer lugar, el sistema se alimenta por información anterior que puede contener errores, que no se evidencia o que no se ha generado desde la fuente, perteneciente a otros Macroprocesos del SGSSS. En otras ocasiones, es el mismo funcionamiento de este Macroproceso el que las origina.

Por esta razón, las categorías que se evidenciaron en el apartado anterior como causantes de información duplicada, también fueron evidenciadas por el equipo de trabajo de esta consultoría como causantes de información equivocada, de que esta información no se reporte oportunamente o no sea evidente o, en el peor de los casos, que esta información no exista o no haya sido generada.

Es decir, también podrían entenderse que las causas explicadas anteriormente asociadas a los riesgos de duplicidad pueden en algún momento causar riesgos de equivocación, inexistencia o de información oculta para actores interesados, situación que puede suceder en atención a la dinámica de los procesos.

Estas causas enunciadas en el apartado anterior tendrían que ver con, entre otras, uso de diferentes fuentes de origen, acceso en diferentes momentos que posibilitan que haya habido transformación de la misma, reporte de la misma información por entidades diferentes que se puede originar en diferentes bases de datos diferentes entre sí pero que debería ser exactamente igual, cambios en la información o categoría de los usuarios que no es depurada y genera registros adicionales en vez de unificarse, existencia de diferentes entes de control que requieren la misma información desde diferentes fuentes que no han sido filtradas, información financiera y/o contable que presenta errores o diferencias no conciliadas y diferencias en la periodicidad con la que se consolida la información en las fuentes y por tanto no es comparable.

Además de lo anterior, existen otras situaciones que pudieron evidenciarse como causantes de estos errores, las cuales se explicarán a continuación.

- Una situación de ausencia de información o de información equivocada se evidenció por parte del equipo de trabajo de esta consultoría tiene que ver con el registro de los nacidos vivos, situación más común en los sectores rurales. Este riesgo existe cuando por la misma idiosincrasia o por ignorancia de la población, el registro de los nacimientos no se hace al no ser considerada como una actividad importante y por tanto se pospone en el tiempo. El mayor riesgo evidenciado en esta categoría se formaliza en el subregistro de la población, o en el registro de información no equivalente en bases de datos de diferente índole. En estos ejemplos se evidencia que la información no corresponde en su totalidad al consultarla en los repositorios de la Registraduría, el Ministerio, las notarías, etc.
- En aspectos relacionados con la materialización del riesgo en salud, e equipo de trabajo de esta consultoría constató el hecho de que en atención a políticas de confidencialidad de los pacientes y de su condición médica, se restringe el acceso a esta información. Sin embargo, algunos actores y algunas etapas de los Macroprocesos necesitan hacer uso de información de manera consolidada y anonimizada y no de manera particular, situación que no infringiría esas políticas de reserva.
- Otra situación que genera posibles errores en la información del SGSSS tiene que ver con la creación y/o cálculo de indicadores de salud que requieren información proveniente de diferentes fuentes. En este caso y en razón a posibles situaciones como las que ya se han relatado en este Macroproceso, alguna o algunas de las fuentes para este cálculo puede presentar información que no es del todo consistente o verificada y en ese caso, el resultado del cálculo del indicador presentaría la misma falencia en sí mismo y en información posterior que utilice sus resultados como insumo. Esta situación puede ocasionar situaciones de subregistro de registro superior al real en la materia asociada a estos indicadores.
- La multiplicidad de fuentes de información ya ha sido destacada en anteriores casos como generadora de riesgos en la información. Esta situación y las posibles inconsistencias en la calidad de los datos originada por este tipo de situaciones, también genera errores por ejemplo al momento del cálculo de la UPC y para Presupuestos Máximos en el Régimen Subsidiado, donde se han detectado diferentes casos de errores y de baja calidad en esta información cuando la fuente de los datos son los prestadores públicos. En estos casos, los niveles de confiabilidad

para diferentes cálculos y actividades se alejan de los estándares aceptables en el Sistema.

- El Sistema tiene una debilidad importante en la gestión de indicadores de oportunidad y de acceso al sistema, donde los cálculos no inician desde el primer intento de un usuario para acceder a alguno de los servicios administrativos o médicos que están contratados, sino que los tiempos y costos empiezan a contabilizarse sólo desde el momento en que este usuario logra acceder al sistema en casos como por ejemplo asignación de una cita médica, entrega de medicamentos o autorización de un procedimiento. En estos casos, si sólo se contabiliza la oportunidad cuando la respuesta fue afirmativa, el indicador siempre será positivo. Pero la realidad del Sistema evidencia innumerables casos en los cuales precisamente las principales quejas de los usuarios temen que ver con la falta de oportunidad y las grandes dificultades para acceder a un flujo de proceso que solucione su situación.
- El equipo de trabajo de esta consultoría evidenció múltiples errores de diferentes tipos en la gestión de las PQRS, algunos de los cuales tienen que ver con prácticas tales como reportar como atención efectiva el envío de respuestas genéricas en todos los casos sin ofrecer solución a los casos particulares pero donde se reporta una gestión efectiva cuando en realidad no lo es, o cuando los tiempos de respuesta se registran desde la última comunicación del usuario antes de lograr la solución requerida pero que ignora los intentos de contacto anteriores que en muchos casos pudieron tardar varios meses.
- En términos generales y en concordancia con el ejemplo anterior en aspectos tales como tiempos de respuesta, asignación de citas, entrega de medicamentos, la información reportada al SISPRO no es totalmente real logrando que se gestionen indicadores positivos para las EPS y las IPS, generando situaciones que no están ajustadas a la realidad y que impiden la toma de correctivos para mejorar el Sistema.
- Como componente de la posible información oculta dentro del Sistema, debe tenerse en cuenta la información que existe pero que sólo se formaliza o se contabiliza en períodos diferentes a los cuales se generó. Esta situación existe por ejemplo en las facturas y en el pago de las mismas, o en indicadores de demoras en la atención donde por medio de alguna figura el registro de esta información se formaliza en períodos posteriores a su ocurrencia, lográndose que el resultado de algunos de estos indicadores no afecte la evaluación de alguno de los actores. En materia contable, esta práctica se evidenció sobre todo tanto en el último como en los primeros meses de cada año, situación que permite la obtención de resultados favorables para algún actor determinado.
- En diferentes situaciones existe el informe no oportuno de algún tipo de información que los diferentes actores deben remitir a los entes de control a pesar de que la normatividad define claramente los tiempos en que estos reportes deben hacerse, es un caso típico de información inexistente, la cual no es generada en este caso por errores procedimentales sino por omisión en alguna de las etapas del Macroproceso en sí mismo o de alguno de los que lo alimenta.

El Macroproceso presenta riesgos de generación de información equivocada, oculta o inexistente cuando la normatividad asociada carece de la suficiente especificidad para ser aplicada en todos los casos y, por lo tanto, permite diferente interpretación de parte de alguno(s) de los actores.

Esta posibilidad también impide la generación de información unificada, teniendo en

cuenta que en algunos casos tampoco existe claridad sobre un único responsable para su generación o creación.

6. Existencia de un consumo innecesario o excesivo de tiempo y/o recursos, ocasionado por los errores identificados.

   Todas las situaciones explicadas anteriormente necesitan un consumo importante de recursos y tiempos para su corrección, teniendo en cuenta que la veracidad y consistencia de la información está asociada a la eliminación de estos errores.

   Este consumo adicional y en la mayoría de los casos innecesario está asociado a aspectos tales como el tiempo necesario para depurar las bases de datos y corregir los resultados erróneos, utilización de recurso adicionales asociados al recurso humano necesario para lograr aclarar y precisar la información.

   En los casos en que diferentes actores necesiten corregir esta información, tampoco existen procesos unificados para lograrlo, por lo que la corrección de errores puede ser abordada de manera simultánea o asincrónica desde diferentes instancias para lograr el mismo resultado. Esta situación que en sí misma genera que se inviertan recursos excesivos para lograr una misma depuración. Esta situación tampoco asegura una corrección adecuada y definitiva dado que mientras no existan procedimientos unificados y existan alertas que identifiquen tanto la información que ya ha sido corregida como la que aún es inconsistente, pueden generarse reprocesos que no aseguran esta formalización.

   De la misma manera, los procesos adicionales de investigación, justificación, verificación y corrección de posibles errores consecuencia de todo lo evidenciado en este capítulo, siempre significarán consumos excesivos e innecesarios de recursos y tiempo para su corrección.

   Los aspectos evidenciados y explicados en los dos apartados anteriores ocasionan situaciones que afectan la calidad y/o completitud de la información, por lo que se requiere que en el momento en que se acude a la misma, se haga necesario adelantar procesos adicionales que no se contemplan en el diseño original de cada proceso para tratar de corregir las posibles inconsistencias en los datos.

   Estas situaciones dificultan los procedimientos asociados a la IVC en el sistema, debido a que existe información duplicada, errada e inoportuna que, sumado a las debilidades en el proceso de supervisión, generan toma de decisiones de manera tardía o basado en hechos y datos no confiables.

7. Contribución de la solución de estos errores al correcto funcionamiento del modelo de gestión del conocimiento.

   La posibilidad de garantizar la consistencia de toda la información que fluye en este proceso no solo disminuye el uso innecesario de recursos necesarios para posibles correcciones, sino que también la unificación de criterios derivados de esta información de la que disponen tanto los prestadores como los prestadores permitiría que los procesos de conciliación de cartera puedan ser armonizados fácilmente, disminuyéndolos costos y logrando acuerdos entre las partes.

   Por otro lado, si el SGC logra consistencia en la información que alimenta y que se deriva de este proceso, también se hace posible que las decisiones que se tomen derivadas de los resultados de los procedimientos de control sean acertadas y solucionen de la mejor manera los posibles inconvenientes surgidos en el Sistema.

   Así mismo, la posibilidad de garantizar la calidad de la información disminuye los riesgos de procedimientos inoperantes y la ralentización del sistema.

La información que se gestiona en este Macroproceso es el insumo necesario para la supervisión a las EPS en materia de habilitación y permanencia que realiza la Supersalud, teniendo en cuenta la necesidad de garantizar el cumplimiento de los requisitos definidos.
